# Lipid Annotator: Towards Accurate Annotation in Non-Targeted Liquid Chromatography High-Resolution Tandem Mass Spectrometry (LC-HRMS/MS) Lipidomics Using a Rapid and User-Friendly Software

**DOI:** 10.3390/metabo10030101

**Published:** 2020-03-12

**Authors:** Jeremy P. Koelmel, Xiangdong Li, Sarah M. Stow, Mark J. Sartain, Adithya Murali, Robin Kemperman, Hiroshi Tsugawa, Mikiko Takahashi, Vasilis Vasiliou, John A. Bowden, Richard A. Yost, Timothy J. Garrett, Norton Kitagawa

**Affiliations:** 1 Department of Pathology, Immunology and Laboratory Medicine, University of Florida, Gainesville, FL 32610, USA; ryost@chem.ufl.edu (R.A.Y.); tgarrett@ufl.edu (T.J.G.); 2 Department of Environmental Health Sciences, Yale School of Public Health, New Haven, CT 06520, USA; vasilis.vasiliou@yale.edu; 3 Agilent Technologies, Santa Clara, CA 95051, USA; xiangdong_li@agilent.com (X.L.); sarah.stow@agilent.com (S.M.S.); mark_sartain@agilent.com (M.J.S.); adithyamurali@gmail.com (A.M.); nkitagawa@gmail.com (N.K.); 4 Department of Chemistry, University of Florida, Gainesville, FL 32611, USA; robin.kemperman@hotmail.com (R.K.); john.bowden@ufl.edu (J.A.B.); 5 RIKEN Center for Integrative Medical Sciences, 1-7-22 Suehiro-cho, Tsurumi-ku, Yokohama, Kanagawa 230-0045, Japan; hiroshi.tsugawa@riken.jp (H.T.); mikiko.takahashi@riken.jp (M.T.); 6 RIKEN Center for Sustainable Resource Science, 1-7-22 Suehiro-cho, Tsurumi-ku, Yokohama, Kanagawa 230-0045, Japan; 7 Center for Environmental and Human Toxicology & Department of Physiological Sciences, University of Florida, Gainesville, FL 32611, USA

**Keywords:** lipidomics, lipid annotation, tandem mass spectrometry, liquid chromatography, metabolomics, ion mobility, metabolomics, automation, software, time-of-flight

## Abstract

Lipidomics has great promise in various applications; however, a major bottleneck in lipidomics is the accurate and comprehensive annotation of high-resolution tandem mass spectral data. While the number of available lipidomics software has drastically increased over the past five years, the reduction of false positives and the realization of obtaining structurally accurate annotations remains a significant challenge. We introduce Lipid Annotator, which is a user-friendly software for lipidomic analysis of data collected by liquid chromatography high-resolution tandem mass spectrometry (LC-HRMS/MS). We validate annotation accuracy against lipid standards and other lipidomics software. Lipid Annotator was integrated into a workflow applying an iterative exclusion MS/MS acquisition strategy to National Institute of Standards and Technology (NIST) SRM 1950 Metabolites in Frozen Human Plasma using reverse phase LC-HRMS/MS. Lipid Annotator, LipidMatch, and MS-DIAL produced consensus annotations at the level of lipid class for 98% and 96% of features detected in positive and negative mode, respectively. Lipid Annotator provides percentages of fatty acyl constituent species and employs scoring algorithms based on probability theory, which is less subjective than the tolerance and weighted match scores commonly used by available software. Lipid Annotator enables analysis of large sample cohorts and improves data-processing throughput as compared to previous lipidomics software.

## 1. Introduction

Lipids are an incredibly complex class of non-polar small molecules with a vast diversity in the number of known lipid species and their biological roles. The entire range of lipids in a given substrate are called the lipidome. The structural and functional diversity of lipids explains the recent spike and continually expanding interest in lipidomics (comprehensive measurement of the lipidome) and includes application in clinical [1,2,3], material [4,5,6], agricultural [7,8], environmental sciences, and many other domains. While new lipids are discovered almost monthly, the complete diversity of lipids is still unknown, even within humans [9]. By increasing the coverage and accuracy of lipid identifications, scientists can better determine biological effects and lipid-based diagnostic markers of disease and other biological perturbations, as well as discover new lipids for novel materials. Though untargeted data-acquisition using liquid chromatography high-resolution tandem mass spectrometry (LC-HR-MS/MS) currently provides a wealth of information on lipids, processing the immense mass spectral data to provide accurate lipid annotations and corresponding relative lipid concentrations remains a challenge.

Since the release of LipidBlast in 2013 [10], there has been a rapid increase in the number of vendor and open-source software solutions for processing mass spectral lipidomics data; currently over 25 software solutions exist [11,12]. Few software cover the entire LC-HRMS/MS lipid data-processing workflow, which includes feature finding, annotation, manual validation, and normalization, with MS-DIAL being the most commonly used open-source software covering the majority of the workflow [13]. The high random-access memory (RAM) and processing speeds required by most current featuring finding and annotation software solutions limit their ability to analyze medium to large LC-HRMS/MS samples sets (i.e., tens to hundreds of samples). Clinical and medical research studies often require large sample sizes, making software performance a limiting factor in the advancement of lipidomics. 

In addition to processing times, accurate annotation is a challenge. The majority of available software employ in silico lipid libraries, which are developed by computing all combinations of fatty acids, backbones, linkages, and head groups, and combining them in all possible arrangements. Fragmentation can be predicted using a simple set of rules extracted from a few lipid standards per class. These libraries can often be rapidly developed, which is ideal as new lipids are continually discovered with automated tools [14,15], and thus this approach allows for the comprehensive lipidomics analysis of fatty acyl-based lipids. However, using this common approach, non-fatty acyl lipids such as sterols and lipophilic vitamins are generally excluded, as their fragmentation spectra are more complex and/or less informative. 

Lipid annotation using in silico libraries often leads to a relatively high false positive rate [16]. False positives often occur due to high spectral impurity (numerous co-isolated lipid precursors for fragmentation) [17], limited lipid standards for simulating MS/MS and validation, and lack of available methods to quantify the false positive rate for any given software or application [18]. In addition, lipid isomers with subtle, but biologically important structural differences co-elute in many cases. Most often the complete lipid structure cannot be characterized by MS/MS alone. For example, evidence of double bond position and branching in fatty acids may not be conferred by most conventional MS/MS systems. Therefore, lipid annotations should reflect only the degree of structural information supported by fragmentation spectral evidence [19]: the nomenclatures in lipidomics are currently suggested in lipidomics standards initiative (LSI; https://lipidomics-standards-initiative.org/). Lipid software solutions often over-annotate, and establishing a common consensus of acceptable protocols is difficult given the large diversity of lipids [20]. Without algorithms for determining the false positive rate, spectral purity, and deconvolution of mixed MS/MS spectra, MS/MS annotations must be validated manually if high confidence in annotations is needed. Manual validation often does not occur in practice and requires extensive knowledge of fragmentation pathways.

In this manuscript, Lipid Annotator is introduced as a user-friendly lipidomics software for Agilent .d files, which can be employed to rapidly analyze large lipidomics LC-(IM)-HRMS/MS datasets and improve the accuracy of annotation. Lipid Annotator employs unique algorithms (non-negative least squares) to deconvolute mixed MS/MS spectra and annotate lipids to the correct degree of structural precision, as supported by MS/MS evidence. Lipid Annotator employs Bayesian statistics, using probability distributions derived from random errors in measurement performance (*m*/*z*, isotopic fidelity, artifacts in MS/MS spectra, and fragmentation signal distributions) in order to approximate the likelihood of individual lipid candidates as well as candidate mixtures to explain the spectra. The application of Bayesian theorem is unique in the field of lipidomics annotation, with the majority of software employing rules based on annotation or weighted scoring schemas, which can be arbitrary and more difficult to interpret than probabilities. It is easier to tune the code to reduce false positives and negatives if Bayesian methods are used through explicit characterization of the error distribution. In addition, Bayesian methods can provide more accurate estimations through the ability to incorporate prior knowledge and direct experimental measurements into error distributions [21]. Software employing Bayesian methods for identification from mass spectra have shown promising results in proteomics [22,23], lipidomics [24], and metabolomics [25]. Application of the Bayesian theorem in lipidomics stands to benefit from better characterization of distributions due to measurement errors (which are instrument and often experiment specific) and real world probabilities, such as those of lipid occurrences in different substrates. 

## 2. Results and Discussion

### 2.1. Lipid Annotator Software

#### 2.1.1. User-Workflow

**Figure 1:**
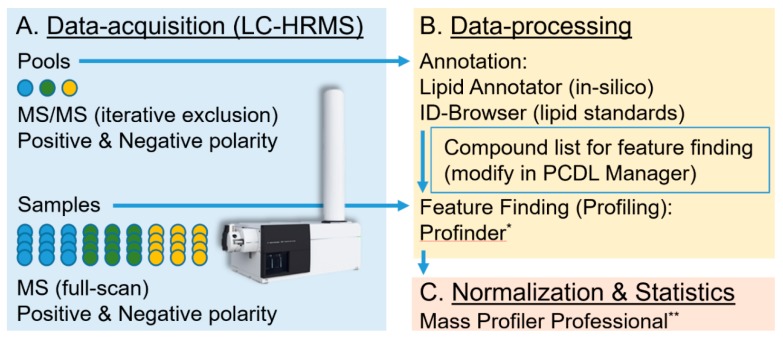
Example of a lipidomics workflow employing Lipid Annotator. MS/MS can be acquired on only a few representative samples saving acquisition and processing time (**A**). Note that iterative exclusion MS/MS of various pools from different groups (e.g., healthy, disease, and control) can be imported into a single Lipid Annotator project, or all groups can be pooled and analyzed via Lipid Annotator. Resulting data is imported into Lipid Annotator (and optionally ID-Browser) to obtain annotations (**B**). Annotation data (including retention times and *m*/*z*) are used to determine features (**B**) across all samples (**A**). Resulting feature tables are imported into mass profiler professional (**C**) to perform normalization (including normalization by lipid internal standards), data-visualization, and multivariate and univariate statistics. Acronyms are defined in Appendix B. * The targeted peak list of annotated lipids *m/z* and retention time can also be used for feature finding via MZMine, XCMS, and other open-source software. ** Resulting data can also be processed via Metaboanalyst and other open-source software.

Lipid Annotator can be used as a standalone tool for the rapid peak picking and annotation of lipids within a given sample, or it can be integrated into a larger LC-HRMS/MS workflow covering all steps, including peak picking, annotation, normalization to lipid internal standards, and statistics. Lipid Annotator is designed only for Agilent LC/ quadrupole time-of-flight (Q-TOF) data files, which limits its scope, but increases the accuracy and simplicity of the software by reducing the need for user parameters and optimization of the parameters and algorithms to Agilent instruments. Figure 1 shows a recommended workflow for comparison of lipid profiles across different groups. Full scan data is acquired for every individual sample (as well as quality controls and extraction blanks). Data-dependent analysis using iterative exclusion is applied to pooled samples of each group to improve MS/MS coverage of lipid ions [26]. 

After annotation in Lipid Annotator and optionally annotating non-fatty acyl lipids using experimental libraries, the final compound list is used for targeted feature extraction across all samples (MS-only data files) in MassHunter Profinder (Agilent). Peaks which do not occur in a large number of samples or which are of too low quality for statistics are filtered out based on user thresholds. The resulting annotated feature table(s) are imported into MassHunter Mass Profiler Professional (MPP, Agilent), where normalization, baselining, median fold changes, constant sums, internal/external scalars, and a wide variety of filtering criteria can be applied prior to statistical analysis. A lipidomics experiment type has been added to MPP to enable annotated lipid analysis. The lipidomics experiment type supports lipid class-based internal standard normalization. Several new visualizations are also supported, included lipid matrix plots (heat maps) at both the lipid species and lipid class level. Additionally, Kendrick mass defect plots and retention time versus mass plots are color coded by lipid class for discernment of class-based trends. 

Lipid Annotator can also be used as the initial annotation step followed by subsequent peak picking and statistical steps using open-source software. Briefly, a text file can be exported from Lipid Annotator which contains the names, mass to charge values, and retention times of annotated lipids, These text files can be formatted as a targeted peak list for peak picking using MZMine 2 [27] (optionally followed by GNPS) or other mass spectral processing software, which can perform numerous steps including chromatogram peak picking, deconvolution, isotopic peak grouping, alignment, gap filling, further library searching and MS/MS similarity scoring [28]. MetaboAnalyst [29] can also be used for downstream statistical analysis either following MZMine 2 by direct export of the peak table file or by exporting and formatting outputs from MassHunter Profinder or MPP.

#### 2.1.2. Lipid Annotator Libraries

Lipid Annotator in silico libraries use MS-DIAL in silico libraries [13] as its source of theoretical fragmentation spectra. MS-DIAL libraries are the most extensive set of in silico MS/MS libraries containing both fragment *m/z* and predicted intensities for Q-TOF MS/MS based approaches and new releases continue to expand the scope of lipid coverage. An algorithm was developed to validate MS-DIAL libraries based on formula prediction of fragments and internal consistency of fragmentation across fatty acyl constituents of a given class. Flagged libraries were removed or corrected, and new libraries were added. Libraries were validated against 63 lipid standards across 21 lipid classes purchased from Avanti Polar Lipids, Inc. and Nu-Chek Prep, Inc. Currently, 58 lipid types are covered when considering all ether and oxidized lipids each as a single lipid type. Within Lipid Annotator, the user can view a table of all in silico lipid libraries by class, by precursor *m/z* match, or by text query similar to LipidPioneer [30]. 

#### 2.1.3. Lipid Annotator Annotation Algorithm

**Figure 2:**
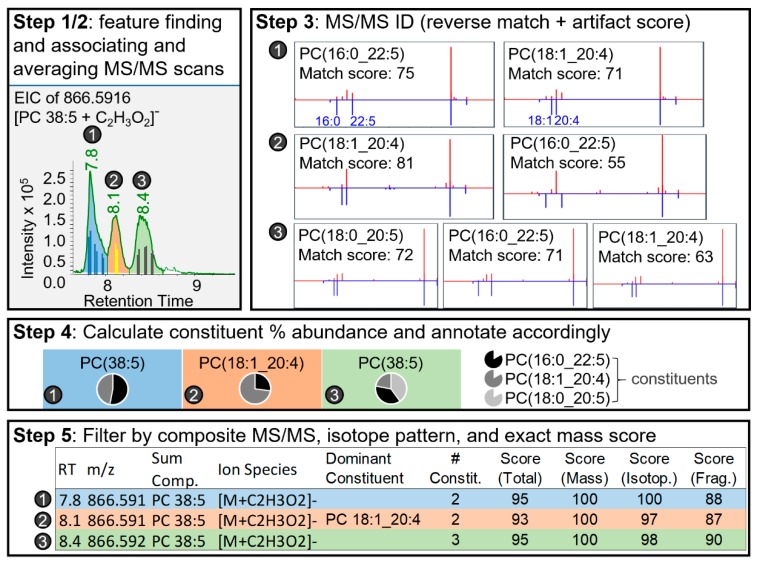
Steps of Lipid Annotator identification algorithm using actual data from National Institute of Standards and Technology (NIST) SRM 1950 human blood plasma acquired in negative polarity. In **Step 1** three chromatographic peaks are integrated and in **Step 2** the MS/MS scans are averaged for each peak. In **Step 3** the average MS/MS are used to identify three possible peak constituents: phosphatidylcholine PC(16:0_22:5), PC(18:1_20:4), and PC(18:0_20:5). In **Step 4** the percent constituents under each peak are calculated using negative least squares fitting of in silico spectra to experimental spectra. In **Step 5** the composite in silico spectra is matched against the experimental spectra and total scores for MS/MS are calculated, which, along with precursor isotopic score and exact mass match scores, can be used to filter results to reduce false positives. Fatty acyl annotation is only provided in Step 5 if the top percent abundance differential between the first and second top most abundant lipid in Step 4 is above a certain threshold. Acronyms are defined in Appendix B.

An in-depth discussion of the Lipid Annotator algorithms, mathematical derivatizations, and theoretical explanations are provided in the Appendix A and supplemental figures. Briefly, the Lipid Annotator algorithm, for annotation based on the in silico libraries, consists of five general steps: feature finding (Figure 2, Step 1), association of MS/MS scans with features (Figure 2, Step 2), annotation of possible lipids for each feature (Figure 2, Step 3), calculation of the percent abundance of each fatty acyl constituent under a single chromatographic peak in the case of mixed spectra (Figure 2, Step 4), and filtration of final annotated features according to exact mass, isotope, and MS/MS match probabilities (normalized to 100) (Figure 2, Step 5). 

Lipid Annotator is used to annotate a feature at two levels. First, algorithms based on the Bayesian theorem [31] are employed to determine which sum composition (sum mixture of lipids for a given class with varying fatty acyl constituents containing the same number of carbons and double bonds) is most likely for a given feature. Essentially, Bayesian probability is used to choose between two potentially overlapping isomers from differing lipid classes, for example, phosphatidylcholine PC(17:0/18:1) and phosphatidylethanolamine PE(16:0/22:1). If multiple lipid isomers co-elute from one lipid class (with differing fatty acyl constituents, in Lipid Annotator referred to as “constituents”), we can estimate the relative abundances of constituents in the mixture by using a non-negative least squares fit (Figure 2, Step 4).

**Figure 3:**
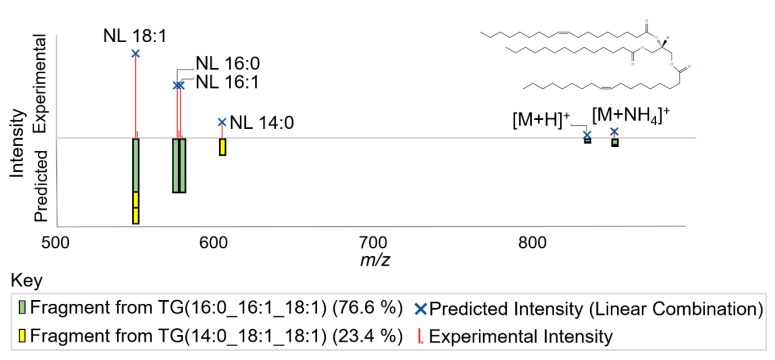
Example of the approximation of percent contribution to a mixed MS/MS spectra of two triglyceride isomers with 50 carbons and 2 degrees of unsaturation, triglyceride TG(50:2), using a negative least squares best fit. Example is from NIST SRM 1950 in positive ion mode.

Non-negative least squares is applied to optimize the abundances of individual lipid ions, in order that their cumulative in silico MS/MS spectral signal best matches the experimental MS/MS spectra (Figure 3). This percent abundance ranking allows the user to evaluate the relative contributions of the different lipid fatty acyl constituents present for a given lipid sum composition. In cases where all constituents have similar percent abundance, the sum composition lipid name is used for downstream analysis. In cases where there is a predominant lipid defined at the level of fatty acyl constituents, the feature is annotated by fatty acyl constituents, which can be used for further biological interpretation (Figure 2, Step 4). The advantage of semi-quantitative determination of lipid abundances under co-eluting chromatograms is shown in Figure 2, Step 4. Whereas 3 peaks are observed, based on pie charts of fatty acyl lipid distributions it can be recognized that there are at least 5 lipid isomers. PC(16:0_22:5) occurs as a higher portion of peak 1 and 3 then peak 2, showing the existence of two deconvoluted isomers of PC(16:0_22:5), which in neither case are the dominant lipids. In this case these species may differ in position of fatty acids on the backbone or positions of double bonds (n-6 DPA and n-3 DPA). Only in the 2nd peak is there a dominant lipid species, which can be reported by fatty acyl constituents for downstream statistics. 

The non-negative-least squares algorithm for deconvoluting mixed spectra (even when chromatograms of isomers completely overlap) has several limitations, which plague any deconvolution algorithm employing data-dependent MS/MS to lipidomics. For example, in silico spectra for which deconvolution depends are imperfect; the effect of fatty acyl chain unsaturation and chain length on fragmentation profiles is not accounted for and instrument conditions used to generate in silico libraries may differ from user conditions. Furthermore, only a single MS/MS scan is required for deconvolution in Lipid Annotator (to improve coverage). This MS/MS scan(s) may not appropriately cover the differing distributions of co-eluting precursor ions, which has previously been discussed [17]. While software has been developed which takes advantage of multiple MS/MS scans to reconstruct co-eluting precursor elution profiles [32], the number of MS/MS scans required for each mass to reconstruct precursor elution profiles would drastically reduce coverage. Therefore, this technique providing percent abundances of co-eluting isomers is qualitative, not quantitative.

The MS/MS spectral match (Figure S1), precursor mass, and isotope pattern (of all combined candidates) of a given feature are each considered independently of one another and multiplicatively contribute to the final probability density of a feature (Figure 2, Step 5). Both independent and final probability densities are used to filter annotated features to reduce false positives. Filter thresholds are user modifiable, with default filters developed to ensure the largest number of false positives are removed and true positives retained using a hand annotated data-set and standards. 

The use of Bayesian theorem and probability densities is unique to Lipid Annotator and provides a more universal approach for annotation based on statistical theory than is currently available in other software. Current lipidomics software approaches uses tolerance windows and/or weighted scoring systems for lipid annotation, which are highly subjective. While these weights can be optimized for a given training data set, weighting schemes lack obvious, logical reasons explaining how the optimization result is applicable to unknown data. The Bayesian method eliminates all weighting schemes. The Bayesian method gives the identification probabilities from, and only from, other than a priori probabilities not dealt with here, measurement distributions, such as ppm errors in *m/z*, signal to noise ratio in fragment spectra, and isotopic ratio fidelity. Certain assumptions must be used in Bayesian methods for lipidomics; mainly that the likelihood of lipid probabilities in a given substrate is unknown (hence the likelihood of each lipid candidate is assumed to be equal), and that the in silico spectra are a good approximate for the actual spectra. In addition, for our purposes, the distribution of errors (e.g., mass error, isotope fidelity, and artifacts in spectra) in most cases were assumed to be normal. One advantage of focusing on a single vendor for software development is that these distributions (e.g., in mass error) can be empirically estimated. Further discussion of assumptions and Bayesian methods is provided in Appendix A.1. 

#### 2.1.4. User Interface and Downstream Workflow

**Figure 4:**
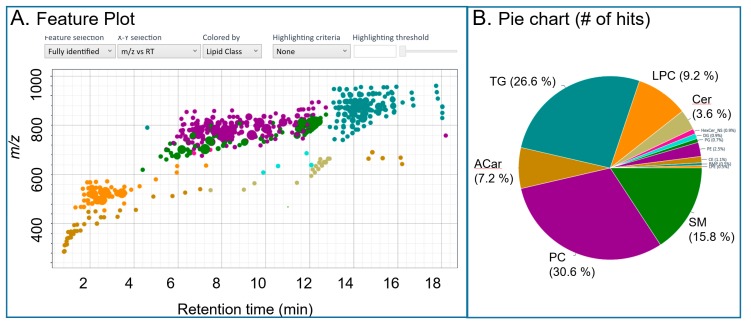
Examples of Lipid Annotator data-visualizations (feature view) for NIST SRM1950 in positive ion mode. Panel (**A**) shows a plots of features which can be used to examine patterns across retention time, mobility, lipid class, identified versus non-identified compounds, Q-score (chromatographic peak quality) and abundance. In panel (**B**) a pie chart displays the total number of annotated lipids per lipid class Note that axis and labels were re-written in larger font to be able to be read in a publication sized figure. Acronyms are defined in Appendix B.

The interface has a feature view (Figure 4) and match details view (Figure S2) for investigating the results. In the feature view, a 2D plot shows each feature detected. The dimensions can be toggled between abundance, retention time, collisional cross sections, drift time (if ion mobility is employed), and *m/z*. The features can be colored by lipid class and highlighted to flag compounds with low chromatographic peak quality (Figure 4). The Match Details view consists of annotated features. Each annotated feature can be selected for further details. Upon selection, the resulting lipid species identified under the same chromatographic peak and their respective match scores, percent abundances, and head-to-tail plots of in silico versus experimental spectra are shown (Figure S3). Based on manual examination of the data, lipids can be removed or added using the MassHunter Personal Compound Database and Library (PCDL) Manager software before further use in downstream analysis. This database is then used to perform a targeted feature extraction from MS1 level data in Profinder based on accurate mass and retention time. This approach improves the speed of data processing by only performing the untargeted peak picking step which is computational expensive on a few representative samples (in Lipid Annotator). It is important to note that because only representative samples are analyzed in Lipid Annotator, the semi-quantitative distributions of lipids determined through non-negative least squares is not passed on into further steps up the workflow, but rather is simply used to assign class based or fatty acyl based annotations. Finally, after the annotations from Lipid Annotator have been aligned with the MS1 data in Profinder, normalization of lipid ions by lipid class can be performed in MPP using user selected internal standards. 

### 2.2. Application and Validation: Analysis of NIST SRM 1950 using Iterative Exclusion 

#### 2.2.1. Lipid Coverage

**Figure 5:**
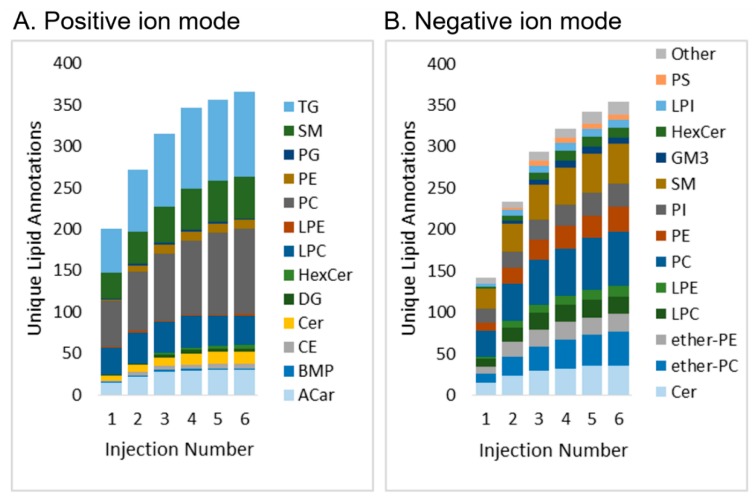
Iterative exclusion in positive (**A**) and negative (**B**) ion mode, showing an increase in the number of annotations over injections when using iterative exclusion. Acronyms are defined in Appendix B.

The workflow presented here uses pooled or representative samples to obtain annotations of features; the annotated features are then used for targeted chromatographic peak detection across all samples, thereby increasing throughput and consistency in peak picking. Because MS/MS is not obtained on all samples, by applying iterative exclusion (IE) for repeated injections of pools, the lower abundant lipid ions can be annotated. In contrast to a prior study [26], iterative exclusion on NIST SRM 1950 human plasma proved more advantageous in negative polarity, with a 149% increase in the number of unique annotations after six injections in negative ion mode as compared to 82% in positive ion mode (Figure 5). This could be due to the higher injection volumes used for negative ion polarity and higher levels of chemical background in this case.

In addition to iterative exclusion, using higher injection amounts (more lipids loaded onto the column) improved annotation due to higher signal for low abundant compounds (Figure S4) and was obtained without significantly affecting mass accuracy of the detected features over a broad range of abundances in the concentrated pooled sample (Figure S5). The presented workflow increases the total number of annotations. In addition, unique databases in Lipid Annotator improved lipid coverage and the precision of annotation. For example, for lysophosphatidylcholine LPC(18:1), multiple isomers were separated chromatographically (four were annotated) and the stereospecific numbering (sn) positional isomers were assigned using the *m/z* 104 fragment, which is unique to fatty acyl chains in the sn2 position of LPC as [M+H]^+^ adducts [33] (Figure S6). The additional isomer(s) could be due to branching in fatty acyl chains or position/stereochemistry of the double bond, which cannot be discerned using traditional MS/MS methods. There were a significant number of lipid isomers whose structural differences could not be resolved by MS/MS. For example, in positive ion mode 19 lipids annotated at the fatty acyl constituent level had one or more identical annotation eluting at a different retention time. Forty-two lipids annotated at the sum composition level (without a predominant fatty acyl constituent, or any fatty acyl fragment information) had one or more identical annotation eluting at a differing retention time. In summary, 17% of the 365 unique lipid species annotated in positive mode had identically annotated isomers, showing the extent of future work needed in routine lipidomics analyses to delineate isomers.

The use of Lipid Annotator on six injections of NIST SRM 1950 using iterative exclusion resulted in 608 unique lipids annotated by Lipid Annotator after combining positive and negative polarity data (class distributions shown in Figure S7 raw data shown in Software_Outputs.xlsx). In this case unique lipids refers to the number of lipid species after combining differing adducts for a single molecular species and combining chromatographically resolved isomers which were indistinguishable by MS/MS. In addition to in silico libraries, experimental MS/MS libraries from over 800 lipid standards were searched against Lipid Annotator. A total of nine unsaturated fatty acids, one branched fatty acid, acetylcarnitine, vitamin E, and cholesterol sulfate were uniquely identified using experimental MS/MS libraries and not annotated using in silico libraries. This shows the advantage of a hybrid in silico–experimental approach for lipidomics to determine both fatty acyl and non-fatty acyl lipids.

Lipid Annotator, LipidMatch, and MS-DIAL annotated 356, 324, and 336 unique lipids in negative ion mode, and 365, 325, and 466 unique lipids in positive ion mode, respectively (Table S4). Total features annotated by each software are included in Table S3. All software outputs can be found in the supplemental excel file.

Lipid Annotator was the software with the highest computational speed (run on a computer with 16 GB RAM, intel Core i7-7700HQ CPU at 2.80 GHz, 64 bit operating system). The software process for annotation (positive mode, 6 IE files, NIST SRM 19560) was less than 1 min as compared to LipidMatch, which took 78 min and MS-DIAL processing which took 8 min. Both of these calculations do not account for file conversion, which is an unnecessary for Lipid Annotator. Further tests across the broader set of lipidomics software and across larger datasets are needed to benchmark the efficiency and speed of Lipid Annotator, but preliminary applications show an advantage in reducing the computational bottleneck in lipidomics workflows.

#### 2.2.2. Annotation Accuracy

To date, one of the major issues with lipid software is the determination of the rate of false positives, due to the difficulty in establishing a decoy database. Therefore, the accuracy of Lipid Annotator annotations was explored through:

(1) internal and external standard solutions,
(2) comparing annotations against other lipidomics software.

Moreover, 11/14 of the Lipidomix deuterated PC, PE, phosphatidylserine (PS), phosphatidylglycerol (PG), phosphatidylinositol (PI), phosphatidic acid (PA), LPC, lysophosphatidylethanolamine (LPE), cholesterol ester (CE), monoglyceride (MG), diglyceride (DG), triglyceride (TG), sphingomyelin (SM), and cholesterol standards spike into the human plasma were correctly annotated by Lipid Annotator, with the exception of CE, DG, and MG, most likely due to the low ionization efficiencies of MG and DG and in-source fragmentation of CE. Of the 86 standards spiked into neat solutions, 63 were correctly annotated by Lipid Annotator (by class, carbons, and unsaturations), 23 were not annotated (mainly due to the lack of libraries to cover them or lack of detection during acquisition), and none were incorrectly annotated at the level of fatty acyl constituents (Lipid_Standards_Info.xlsx).

**Figure 6:**
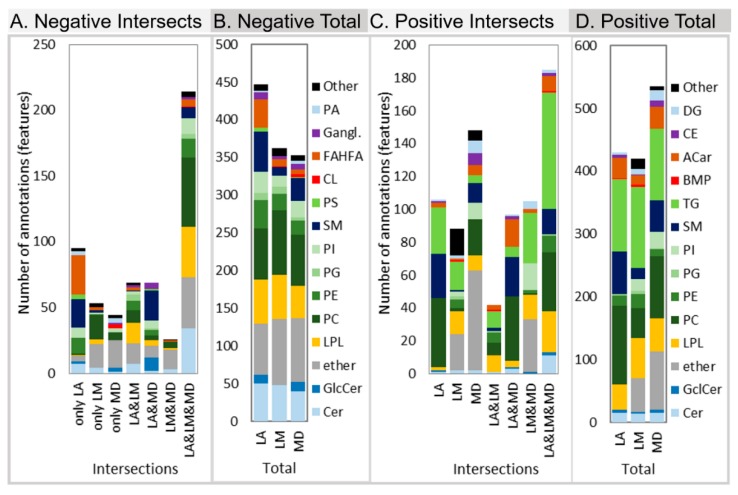
Comparison of annotations of features across the untargeted lipid annotation software Lipid Annotator (LA), LipidMatch (LM), and MS-DIAL (MD). Bars are color coded by lipid class. Bar graphs (**A**) and (**C**). represent the lipid annotations, which were only determined in one software (only) and those which were annotated in two or more software (annotation intersections denoted by “&”), for negative and positive mode, respectively. Bar graphs (**B**) and (**D**) represent the total annotations for each software individually divided by lipid class for negative and positive mode, respectively. Prior to this analysis both internal standards and skin ceramides were removed. Note fatty acid esters of hydroxy fatty acids (FAHFA) was removed from analysis due to likely false positives (see text). Acronyms are defined in Appendix B.

Lipid Annotator, LipidMatch, and MS-DIAL annotation of the human plasma samples were compared (Figure 6) (Note that in this case total features annotated are compared (not unique lipid annotations), and therefore numbers are greater in Figure 6 than Figure 5 for Lipid Annotator). Each software uses unique algorithms for annotation. In LipidMatch, specific lipid fragment *m/z* values must be observed for confirmation (class-based rules), and summed fragment intensities are used to rank co-eluting lipid isomers. MS-DIAL uses a weighted scoring algorithm which includes modified reverse dot product scoring for MS/MS, isotopic distribution scores, and rules to determine at what level of structure to annotate in a manner similar to LipidMatch. Lipid Annotator uses probability density calculations for isotopic pattern, MS/MS spectra, and precursor mass to annotate lipids, and a non-negative least squares fit to determine percent contribution of isomers to a peak.

A comparison of each lipid software with different algorithms for annotation produced similar results, suggesting that MS-DIAL, LipidMatch, and Lipid Annotator have a low false positive rate for annotations at the level of sum compositions (Table S5). Moreover, 98% of features with annotations across all software in positive mode (of 176 comparable features) and 96% of features in negative mode (of 132 comparable features) were annotated the same at the level of lipid class, total fatty acyl carbons and level of unsaturation, while 65% and 79% had matching top hits at the level of fatty acyl constituents in positive and negative mode, respectively. Lipid Annotator annotations were confirmed by at least one other software at the level of carbons and unsaturations for 100% and 99% of comparable features, in positive and negative mode respectively, and by fatty acyl constituents for 84% and 88% of comparable features, in positive and negative mode, respectively (Table S5).

All three software platforms generated high agreement using differing algorithms for annotation. This includes the top ranked fatty acyl constituent for a feature being confirmed over 80% of the time by other software, suggesting that the novel algorithm for deconvoluting mixed MS/MS spectra in Lipid Annotator generates an accurate ranking of lipid isomers for a high proportion of annotated chromatographic peaks. Of the three software platforms compared, Lipid Annotator is the only software to estimate the actual levels of each isomer in a mixed MS/MS spectra (Figure 2 and Figure 3). A current limitation to this technique is that the percent contribution of each lipid isomer to an MS/MS scan may not represent the exact percent contribution of each lipid isomer to a chromatographic peak [17]. Briefly, if only a few MS/MS events occur across a chromatographic peak, and the positions of these scans do not accurately reflect the distribution of isomers under the chromatographic peak, then this will skew any quantitation of isomers using MS/MS. Therefore, advances in data-acquisition methods, for example developing scanning methods, which include four or more MS/MS events distributed evenly across the peak, would better represent isomer abundances using MS/MS. In addition, if in silico libraries do not correctly predict experimental MS/MS fragmentation, then percent abundance calculations will be incorrectly estimated. However, this issue can be overcome through improvements in in silico libraries specific to a set collision energy, Q-TOF instruments, and accounting for the effect of unsaturations and carbons on ionization efficiencies.

A more in-depth analysis of features annotated after removing internal standards can be seen in Figure 6. The proportion of features annotated by all three software platforms was higher in negative mode (Figure 6A) than in positive ion mode (Figure 6C). Hence, there are more discrepancies in annotation between software packages in positive ion polarity data. This is further verified by the fact that of those features with annotations across all three software platforms, there was better agreement of annotations in negative polarity than positive polarity as described above. In negative ion mode, Lipid Annotator had the most lipids which were verified by one or more other software (Figure 6A), the most unique lipids (Figure 6A), and the highest number of total features annotated (Figure 6B). MS-DIAL had the most unique lipids in positive polarity.

It is important to note that in-source fragmentation and solvent clusters can lead to MS/MS spectra identical to precursor ions [34], which are not of biological origin and hence can be considered false positives (see Appendix A). These cannot be discerned without orthogonal approaches including retention time or ion mobility separation, and/or prior knowledge/expert review. For example, the cluster ions incorporating solvent and fatty acids can be misannotated as fatty acid esters of hydroxy fatty acids (FAHFAs): we excluded the annotation of FAHFAs from the original output in this study because they are rarely detected in human serum with our conventional LC-MS method. While these were considered false positives, these species have been detected in plasma previously [35], and therefore using prior knowledge we reduce false positives while potentially limiting the discoveries of novel lipids, or known lipids previously unknown to exist within a biological compartment/fluid. Other compounds which are products of ionization mechanisms rather than of biological origin, for example the in-source fragment lyso-lipids originating from their precursor phospholipids, are also often annotated when solely using MS/MS as annotation criteria [34], but will elute at the retention times of the precursor not of their analyte counterparts. Therefore, including retention time (supported in MS-DIAL for example), ion mobility, or other orthogonal separation method in annotation will reduce false positives, and future implementation in Lipid Annotator would be advantageous. One difficulty is that in silico retention time libraries are column and gradient specific and, therefore, collisional cross-sectional values (CCS), which are fundamental properties of ions and hence universal could be more widely adopted. As in all current lipidomics non-targeted software, annotations should be validated by expert review before being 100% confident in the annotation.

## 3. Materials and Methods

### 3.1. Methods: Lipid Extraction and Data-Acquisition

Aliquots (40 µL for (positive mode) and 120 µL for (negative mode)) of thawed plasma (NIST SRM 1950 Metabolites in Frozen Plasma, Sigma, St. Louis USA) were each extracted using a modified Folch extraction procedure [36] and reconstituted in 100 µL of a methanol/chloroform mixture (9:1, *v*/*v*). LC separation was performed on an Agilent 1290 Infinity II LC System, with a 19 min gradient time on a reverse phase C18 column (Agilent InfinityLab Poroshell 120 EC-C18, 3.0 × 100 mm, 2.7 µm). Mobile phase consisted of 10 mM ammonium acetate and 0.2 mM ammonium fluoride in 9:1 water/methanol, while mobile phase B consisted of 10 mM ammonium acetate and 0.2 mM ammonium fluoride in 2:3:5 acetonitrile/methanol/isopropanol. Negative and positive polarity data was acquired on the Agilent 6546 LC/Q-TOF using iterative MS/MS acquisition mode on 6 injections of extracted plasma for each polarity [37]. Detailed experimental methods for chromatography and mass spectrometry can be found in Supplemental Table S1 and Table S2, respectively, and in the Agilent application note 5994-0775en [37]. Two methods were used, a high-load and a low-load method, to determine the effect of high injection volumes/concentration on the number of annotations using the Agilent 6546 LC/Q-TOF.

### 3.2. Methods: Data-Processing

Iterative MS/MS acquisition data of NIST SRM 1950 in positive and negative polarity were separately analyzed by each lipidomics software platform (LipidMatch Flow, MS-DIAL, and Lipid Annotator). Data processing parameters can be found in Appendix A. Resulting annotations from all software were appended to the Lipid Annotator feature table using an R script available in the LipidMatch software package [17]. The FAHFA class was also excluded from the list because the molecules are not detected in our extraction and LC-MS conditions.

## 4. Conclusions

Lipid Annotator can be used on large datasets for rapid annotation, relative quantification, and statistics (using a downstream workflow with MassHunter Profinder and MassHunter Mass Profiler Professional software). In addition to the correct annotation of spiked internal standards, annotations of NIST SRM 1950 were comparable across lipid software using differing annotation algorithms suggesting low false positive rates. As compared to other software, Lipid Annotator contains unique algorithms to deconvolute mixed MS/MS spectra from co-eluting lipid isomers, determines the percent abundance of each lipid isomer contributing to the mixed spectra, and annotates by fatty acyl constituents only if there is a dominant lipid species. Lipid Annotator also is the only software to use probability theory for annotation (which is less subjective than current approaches) and supports ion-mobility data workflows. While Lipid Annotator provides unique algorithms for annotation of lipids, a number of assumptions in library generation and annotation exist, and as with any software, expert review is required prior to 100% confidence in annotations. Future work developing algorithms to determine software false positive, true positive, false negative, and true negative rates would be helpful for users to distinguish quality lipidomics software from that which produces many erroneous annotations or has low coverage. In addition, ground truth lipidomics datasets with manually curated annotations (possibly with the aid of software) for validating and benchmarking lipid annotation algorithms are needed.

## Supplementary Materials

The following are available online at https://www.mdpi.com/2218-1989/10/3/101/s1, **Lipid_Standards_info.xlsx:** Contains a list of standards and false positives, true positives, and false negatives using Lipid Annotator. **Software_Outputs.xlsx:** Processed features tables with lipid annotations from MS-DIAL, LipidMatch, and Lipid Annotator, as well as a combined table of annotations from all 3 software with statistics on agreement between the software. **Raw data is available at:** https://massive.ucsd.edu/ProteoSAFe/static/massive.jsp, MassIVE ID: MSV000084849; https://massive.ucsd.edu/ProteoSAFe/dataset.jsp?task=a18ac48abb6b4148a41a1ec31e86abb3; Figure S1: A figure describing the algorithm for determining MS/MS score. Note that two “artifact peaks” actually come from PC(18:1_20:4) and hence reduce the score due to mixed spectra. Therefore, after negative least squares is applied, the in silico mixed spectra is used to obtain a new MS/MS score, which takes into account co-eluting lipids. *A normalization factor is applied to have a scoring scale between 0–100, Figure S2: Match Details view, showing two panels for the annotation of TG(16:0_16:1_18:1) and Cer(d18:1/18:0), Figure S3: Lipid constituents table (**A**), showing two triglycerides (TG) annotated for the same feature (**B** and **C**) and their percent abundance score, Figure S4: A lipid dense region of the mass spectrum (*m*/*z* 750–765) without (**A**) and with (**B**) peak saturation. The figure shows that lower abundant ions can be observed when the sample is concentrated, which would not have been observed otherwise, Figure S5: Mass accuracy versus abundance (intensity) of ions. Mass accuracy is consistently within 5 ppm on the Agilent 6546 even when peaks are saturated, Figure S6: EICs for LPC(18:1) (5 ppm tolerance) showing 4 annotated isomers. Note the last peak could be an erroneously detected LPC due to peak tailing. The *m*/*z* 104 fragment is used to discern LPC sn1 and sn2 isomers, Figure S7: Distribution of unique lipids by class after combining positive and negative polarity lipid annotation using Lipid Annotator (as a percent of annotations, not intensity or relative amounts). Other consists of one CL and SHexCer, Table S1: UHPLC parameters, Table S2: Mass spectrometric parameters for the Agilent 6546 LC/Q-TOF; Table S3: Total number of features annotated with iterative exclusion data-dependent analysis (IE-DDA), DDA, and IE-DDA with high injection volume; Table S4: Total number of unique lipids annotated per software and polarity. In this case, unique lipids refers to the number of lipid species after combining differing adducts for a single molecular species and combining chromatographically resolved isomers, which were indistinguishable by MS/MS, Table S5: Percent of features which have annotations across all software (excluding ether-lipids) with one or more additional software proving the same annotation at the level of lipid class, carbons and unsaturations (C:DB), and at the level of fatty acyl constituents (FA). “All” represents the percent of feature where all three software provided the same annotation.

## Appendix A

### Appendix A.1. Lipid Annotator Algorithms

Since neither databases nor observed data are error free (due to background noise, simplification of theoretical databases, etc.), lipid annotations for a given feature using precursor and fragment mass spectrometric (MS) peaks are tentative. In addition, frequently a single feature consists of multiple isomeric lipid ions. For these reasons, we annotate a feature at two levels. First, we determine which sum composition (mixture of lipids for a given class with varying fatty acyl constituents containing the same total number of carbons and double bonds) is likely to be the feature based on probability (Bayesian theorem). Then, we calculate the relative abundances of lipids in the mixture which give the best fit to the data.

#### Appendix A.1.1. Sum Composition Annotation Using Bayesian Theorem

Mass spectrometry, along with chromatography and ion mobility spectrometry, provide very rich, multiple-dimension data for the separation and annotation of lipids and other chemical compounds. It is highly desirable to take advantage of all the available data during annotations. Conventional techniques use tolerance windows for matching. For example, a software user may specify a 10 ppm tolerance for the mass and 0.5 min tolerance for retention time. Under this premise, all of the lipids falling into the windows described above are assigned to the feature. More sophisticated software packages allow the user to specify a weighting scheme to combine the fitting of all the dimensions into a single ranking score. Weighting schemes enable discriminative power to determine which annotations are most accurate. Such schemes are highly subjective. While these weights can be optimized for a given training data set, weighting schemes lack obvious, logical reasons as to why the optimization result is applicable to the unknown data.

Here, we apply the Bayesian Theorem [31], which is arguably the most fundamental statistics theory in machine learning to handle classification problems, to our annotation problem. Bayesian Theorem can be summarized as:

(1) $$P\left(w_{i}|x\right)=\frac{p\left(x|w_{i}\right)P\left(w_{i}\right)}{p\left(x\right)},$$

where is the probability that a given feature belongs to class $w_{i}$ (sum composition, in this case) for the given observed data x, $p\left(x|w_{i}\right)$ is the probability density that the data x can be observed for a given sum composition $w_{i},\;P\left(w_{i}\right)$ is a known *a priori* probability of the sum composition $w_{i}$ independent of the data x, and p(x) is the total probability density function given by

(2) $$p\left(x\right)=\sum_{i}\;p\left(x|w_{i}\right)P\left(w_{i}\right),$$

with the sum being over all the possible classes $w_{i}$.

Since we do not, at this stage, have a database giving the universal natural frequency of lipid sum composition occurrences in different substrates, we treat all the lipids equally ($P\left(w_{i}\right)=1$). Namely, we let the observed data be the sole judge to decide the annotation. Using this simplification, we focus on the calculation of $p\left(x|w_{i}\right).$

$p\left(x|w_{i}\right)$ is the probability density of data taking a value of x given sum composition $w_{i}.$ Currently we use precursor masses, precursor isotope patterns, and fragment spectra as our data x. It would be straightforward to include retention times, mobility drift times, and other measurements into the probability calculation upon required databases becoming available. One assumption is that different dimensions of data are statistically independent to one another. Under this assumption, $p\left(x|w_{i}\right)$ can be decomposed into:

(3) $$p\left(x|w_{i}\right)=\prod_{j}p\left(c_{j}|w_{i}\right),$$

where indexes *j* run over the dimensions (precursor mass, isotope pattern, and MS/MS), and $p\left(c_{j}|w_{i}\right)$ is the probability density for $w_{i}$ taking value $c_{j}$.

In the following, we give some detailed examples to illustrate how we calculate these $p\left(c_{j}|w_{i}\right)$ in practice. For precursor mass, for any sum composition, we can calculate its theoretical mass $m_{0}$. If we assume its observed value m has a Gaussian distribution, we can write, up to a scaling factor,

(4) $$p\left(m|w_{i}\right)=e^{-{\left(m-m_{0}\right)}^{2}/2\sigma^{2}},$$

where σ is a constant for a give instrument and data acquisition protocol and is obtainable empirically.

For fragment spectra matching, a common traditional technique involves “forward” and “reverse” searches using dot-products of library and observed spectra [10,13,32]. This heuristic approach does not fit our theoretical framework. As a new approach, we assume the data deviation from the database values are due to two independent factors: impurity and mismatch (Figure S1). The impurity covers all the observed peaks not associated with database peaks (e.g., background noise), and the mismatch describes the difference in intensity patterns between database peaks and observed peaks. Two observables (or derivatives from observations) are $I=\sqrt{\sum_{i}h_{i}^{2}⁄H_{D}}$ and $M=\sqrt{\sum_{d}{\left(h_{d}-{\hat{h}}_{d}\right)}^{2}⁄H_{D}}$ where $h_{i}$ are intensities of impurity peaks, $h_{d}$ are intensities of experimental peaks associated with database peaks, ${\hat{h}}_{d}$ are peak intensity values from the database, and $H_{D}=\sum_{d}{\hat{h}}_{d}^{2}$. Thus, the probability density of fragment spectra $p\left(f|w_{i}\right)$ of $w_{i}$ becomes $p\left(f|w_{i}\right)=p\left(I|w_{i}\right)$ $p\left(M|w_{i}\right)$ where $p\left(I|w_{i}\right)$ and $p\left(M|w_{i}\right)$ are the probabilities of $w_{i}$ taking values *I* and *M*, respectively. The distributions of $p\left(I|w_{i}\right)$ and $p\left(M|w_{i}\right)$ can be obtained either by doing statistics on data from known lipids, or simply by heuristics. Note that the exact form of *I* and *M* are not crucially important, as long as we have reasonably good models of their statistical distributions.

Under the assumption that both *I* and *M* follow Gaussian distributions, we can write the probability density of the fragmentation data as

(5) $$p\left(f|w_{i}\right)=e^{-I^{2}/2\sigma_{I}^{2}}\;e^{-M^{2}/2\sigma_{M}^{2}},$$

where constants $\sigma_{I}$ and $\sigma_{M}$ are obtained empirically.

In these descriptions, we assume an in silico MS/MS fragmentation for a sum composition exists. If the sum composition is made up of multiple fatty acyl species, then no in silico (predicted) MS/MS library of the sum composition is available in the database. The database only contains individual fatty acyl species in silico MS/MS (e.g., Figure 2, Step 3). Therefore, first, we use the procedures described above to choose candidates of individual lipids, for which fragment spectra are available in the database. Then, we proceed to determine relative abundances of lipids belonging to the same sum composition as described in the next section. Once these relative abundances are available, a composite reference spectrum (as shown in Figure 3) can be calculated for the sum composition, and then a $p\left(f|w_{i}\right)$ for the observation *f*.

It is important to note that deconvolution of overlapping isotopic patterns is performed during molecular feature extraction in Lipid Annotator, reducing contamination of isotopic envelopes. This improves isotopic matches. When the separations in both the retention time and mass domains are not enough for deconvolution, overlapping isotope envelopes do affect the isotope scoring, which does not have as great discriminative power as the mass. One of the most common situation is when two lipids are separated by 2 Da, e.g., PC(36:3) and PC(36:2). In such a case, the lower-mass lipid has only 2 peaks which can be used. However, this limitation is minimal since any higher order peaks are very small for lipids. The effect on the higher-mass lipid could be more severe. However, the contamination from the lower-mass lipid is more damaging to the absolute heights than to the height ratios which are used in the scoring. Overlapping isotopic patterns are less common in reverse phase chromatography, but are more common in hydrophilic interaction liquid chromatography HILIC approaches.

#### Appendix A.1.2. Calculation of Lipid Relative Abundances to Fit Data using Non-Negative Least Squares Fit

Lipid analysis presents an additional challenge compared to metabolite analysis in that often a single feature (precursor mass and retention time and/or ion mobility drift time) consists of multiple overlapping lipid isomers. This algorithm uses a non-negative least squares fit to determine the percent abundance for each lipid as compared to the total feature ion signal (Figure 2, Step 4). Non-negative least squares is applied to optimize the abundances of individual lipid ions, in order that their additive in silico MS/MS spectra best match the experimental MS/MS spectra (Figure 3). This percent abundance ranking allows the user to evaluate the different lipid fatty acyl constituents present for a given lipid sum composition. In cases where all constituents have similar percent abundance, the sum composition lipid name is used for downstream analysis. However, in cases where there is a predominant lipid defined at the level of fatty acyl constituents, the feature is annotated by fatty acyl constituent which can be used for further biological interpretation (Figure 2, Step 4).

After following the steps in Section 3.1., if multiple lipids of the same sum compositions are assigned to a feature, than, letting {$a_{1,\;}a_{2,\;}\ldots a_{N\;}$} represent relative abundances of each fatty acyl constituent and {$s_{1,\;}s_{2,\;}\ldots s_{N\;}$} represent the in silico database spectra of each fatty acyl constituent, we can have a composite spectrum for the sum composition $\hat{s}$:

$\hat{s}=\sum_{i}a_{i}s_{i}$ in a schematic manner. Treating {$a_{1,\;}a_{2,\;}\ldots a_{N\;}$} as unknowns, we can solve the minimization problem for these unknowns:

$$min{\left|\left|s-\hat{s}\right|\right|}^{2}$$

where **s** is the observed data. Since {$a_{1,\;}a_{2,\;}\ldots a_{N\;}$} cannot be negative number, the minimization turns to solving a non-negative least squares equation.

#### Appendix A.1.3. Normalization of Probabilities

As an addition to the annotation algorithm itself, we would like to point out that $p\left(c_{j}|w_{i}\right)$ can be used, after some modification, as a score to indicate how lipid $w_{i}$ fits a particular dimension *j* of the data. The value of $p\left(c_{j}|w_{i}\right)$ has certain unit with it. For example, the unit is 1/Dalton for mass dimension. To build a user-friendly, universally comparable scoring system, we define the score in *j^th^* dimension as $s_{j}=n_{j}p\left(c_{j}|w_{i}\right)$ where $n_{j}$ is a normalization factor such that the best possible fit has a score 100. We can also extend the scoring to a combination of dimensions or to the overall data set by averaging scores of individual dimensions involved. Since the combination of probabilities of individual dimensions follows the multiplication rule (Equation (3)), a natural rule for the averaging is the geometrical average: $\sqrt[{N}]{\prod_{j}s_{j}}$. For example, the score of fragment fit is $\sqrt{s_{I}s_{M}}$ according to the discussion in Section 1, where $s_{I\;}\;and\;s_{M}$ are the scores of the impurity and mismatch, respectively.

#### Appendix A.1.4. User Interface and Downstream Workflow

Screen shots from the interface can be seen in Figures S2 and S3 and in Figure 4. The interface has a feature view and match details view for investigating the results. In the feature view, a 2D plot shows each feature detected. The dimensions can be toggled between abundance, retention time, and *m/z* and the features can be colored by lipid class (Figure 4) or sample. On the right panel, a pie chart shows the number of features annotated for each lipid class. A table consisting of each feature and respective information including retention time, *m/z*, detection across samples, and Q-Score, is shown. In the match details view, a table with lipid annotations, match scores, formulas, adducts, and other pertinent information for identification is provided. Each annotated feature can be selected and the resulting lipid species identified under the same chromatographic peak and their respective match scores, percent abundances, and head to tail plots of in silico versus experimental spectra can be viewed. Based on manual examination of the data, lipids can be removed or added using PCDL Manager before further downstream analysis.

After annotation using Lipid Annotator and peak picking using Agilent Profinder software, normalization of lipid ions by lipid class can be performed in Agilent Mass Profiler Professional software using user selected internal standards. The sample-wise normalization algorithm normalizes all lipids within a class to the internal standard(s) sharing the same lipid class. In the case of multiple internal standards per class, the average signal across internal standards is used for normalization. When an analyte of a specific lipid class has no matching internal standard or the analyte is unidentified, the average signal across all internal standards is used.

### Appendix A.2. Software Settings

#### Appendix A.2.1. MS-DIAL Parameter Setting

MS-DIAL version 3.66 was used (http://prime.psc.riken.jp/) by the following parameters: retention time begin, 0 min; retention time end, 100 min; mass range begin, 0 Da; mass range end, 5000 Da; accurate mass tolerance (MS1) tolerance, 0.01 Da; MS2 tolerance, 0.025 Da; maximum charge number, 2; smoothing method, linear weighted moving average; smoothing level, 3; minimum peak width, 5 scan; minimum peak height, 5000 in positive ion mode and 3000 in negative ion mode; mass slice width, 0.1 Da; sigma window value, 0.5; MS2Dec amplitude cut off, 0; exclude after precursor, true; keep isotope until, 0.5 Da; keep original precursor isotopes, false; exclude after precursor, true; retention time tolerance for identification, 100 min; MS1 for identification, 0.01 Da; accurate mass tolerance (MS2) for identification, 0.05 Da; identification score cut off, 80%; using retention time for scoring, true; relative abundance cut off, 0; top candidate report, true; retention time tolerance for alignment, 0.05 min; MS1 tolerance for alignment, 0.015 Da; peak count filter, 0; adduct ion setting, [M+H]^+^, [M+NH_4_]^+^, [M+Na]^+^, [M-H_2_O+H]^+^, [M-C_6_H_10_O_5_+H]^+^, [2M+H]^+^, [2M+NH_4_]^+^, [2M+Na]^+^ in positive ion mode and [M-H]^−^, [M-H_2_O-H]^−^, [M+CH_3_COO]^−^, [M+Na-2H]^−^, [M-C_6_H_10_O_5_-H]^−^, [2M-H]^−^, [2M+CH_3_COO]^−^ in negative ion mode. Lipid annotations were automatically performed. The following lipid classes were excluded in annotation pipeline to provide the same chemical space as that of LipidAnnotator and LipidMatch: MGDG, DGDG, LDGTS, DGTS, LDGCC, DGCC, SQDG, GlcADG, AcylGlcADG, Cer-EOS, and HexCer-EOS in positive ion mode, and GlcADG, AcylGlcADG, MGDG, DGDG, EtherMGDG, SQDG, Cer-OS, Cer-AS, Cer-ADS, Cer-BS, Cer-BDS, Cer-NP, Cer-EOS, Cer-EODS, HexCer-EOS, Ac2PIM1, Ac2PIM2, Ac3PIM2, Ac4PIM2, and LipidAPP in negative ion mode.

#### Appendix A.2.2. LipidMatch Parameter Setting

LipidMatch was applied with an *m/z* window for matching fragment ions of 10 ppm, a retention time window for assigning MS/MS scans for a feature of 0.3 min, and a minimum number of “scans” with necessary fragments set to 1. All libraries were queried except for those excluded above in the MS-DIAL parameters settings. Formate adducts were excluded from searching. The feature table used as an input for LipidMatch was the same feature table generated by Lipid Annotator, including features without annotations.

## Appendix B List of Acronyms

**Acronym**	**Definition**
Acar	acylcarnitine
BMP	bis(monoacylglycero)phosphate
CCS	collision cross section
CE	cholesterol ester
Cer	ceramide
CL	cardiolipin
DG	diglyceride
EIC	reconstructed ion chromatogram
ether	plasmenyl/plasmanyl lipid
FAHFA	fatty acid ester of hydroxyl fatty acid
Gangl	ganglioside
GlcCer	glucosyl ceramide
GM3	monosialodihexosylganglioside
HexCer_NS	hexosyl-ceramide
HRMS	high resolution mass spectrometry
ID	identification
LA	Lipid Annotator
LC	liquid chromatography
LM	LipidMatch
LPC	lysophosphatidylcholine
LPE	lysophosphatidylethanolamine
LPI	lysophosphatidylinisitol
LPL	lysophospholipid
M	molecular ion
MD	MS-DIAL
MG	monoglyceride
MPP	mass profiler professional
MS/MS	tandem mass spectrometry
NIST	National Institute of Standards and Technology
NL	neutral loss
PA	phosphatidic acid
PC	phosphatidylcholine
PCDL	Personal Compound Database and Library
PE	phosphatidylethanolamine
PG	phosphatidylglycerol
PI	phosphatidylinositol
PS	phosphatidylserine
Q-TOF	quadrupole time of flight
RAM	random access memory
SM	sphingomyelin
SRM	standard reference material
TG	triglyceride
