# Supplementary material for: Lipid Annotator: Towards Accurate Annotation in Non-Targeted Liquid Chromatography High-Resolution Tandem Mass Spectrometry (LC-HRMS/MS) Lipidomics Using a Rapid and User-Friendly Software
Source: Metabolites. 2020 Mar 12;10(3):101. doi: 10.3390/metabo10030101 (PMC7142889; doi:10.3390/metabo10030101)
Supplement: Supplementary file 1 [file metabolites-10-00101-s001.zip › LipidAnnotator_Figures&Tables_Supplemental.pptx]

## Slide 1
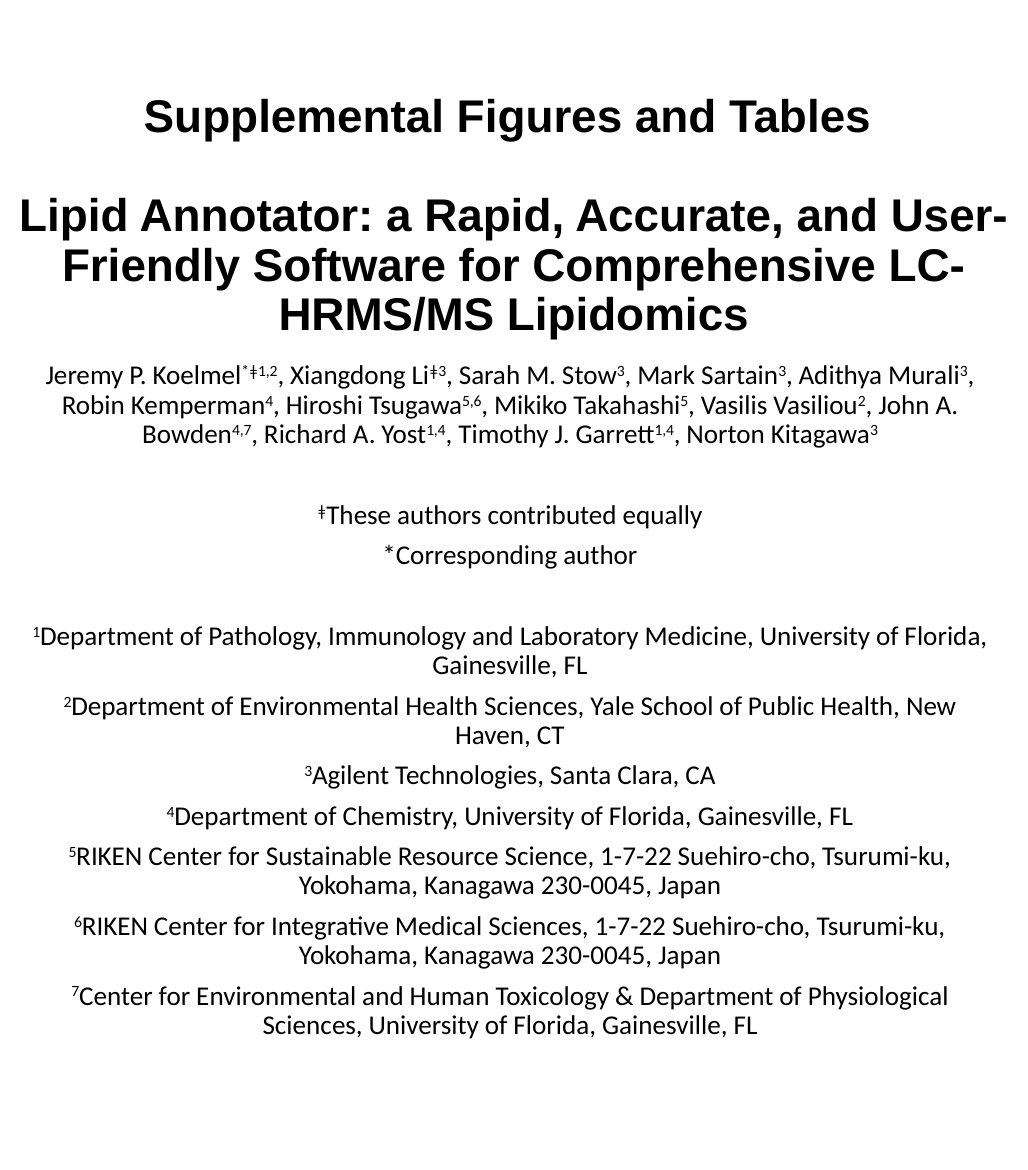

# Supplemental Figures and Tables Lipid Annotator: a Rapid, Accurate, and User-Friendly Software for Comprehensive LC-HRMS/MS Lipidomics
Jeremy P. Koelmel*ǂ1,2, Xiangdong Liǂ3, Sarah M. Stow3, Mark Sartain3, Adithya Murali3, Robin Kemperman4, Hiroshi Tsugawa5,6, Mikiko Takahashi5, Vasilis Vasiliou2, John A. Bowden4,7, Richard A. Yost1,4, Timothy J. Garrett1,4, Norton Kitagawa3
ǂThese authors contributed equally
*Corresponding author
1Department of Pathology, Immunology and Laboratory Medicine, University of Florida, Gainesville, FL
2Department of Environmental Health Sciences, Yale School of Public Health, New Haven, CT
3Agilent Technologies, Santa Clara, CA
4Department of Chemistry, University of Florida, Gainesville, FL
5RIKEN Center for Sustainable Resource Science, 1-7-22 Suehiro-cho, Tsurumi-ku, Yokohama, Kanagawa 230-0045, Japan
6RIKEN Center for Integrative Medical Sciences, 1-7-22 Suehiro-cho, Tsurumi-ku, Yokohama, Kanagawa 230-0045, Japan
7Center for Environmental and Human Toxicology & Department of Physiological Sciences, University of Florida, Gainesville, FL

## Slide 2
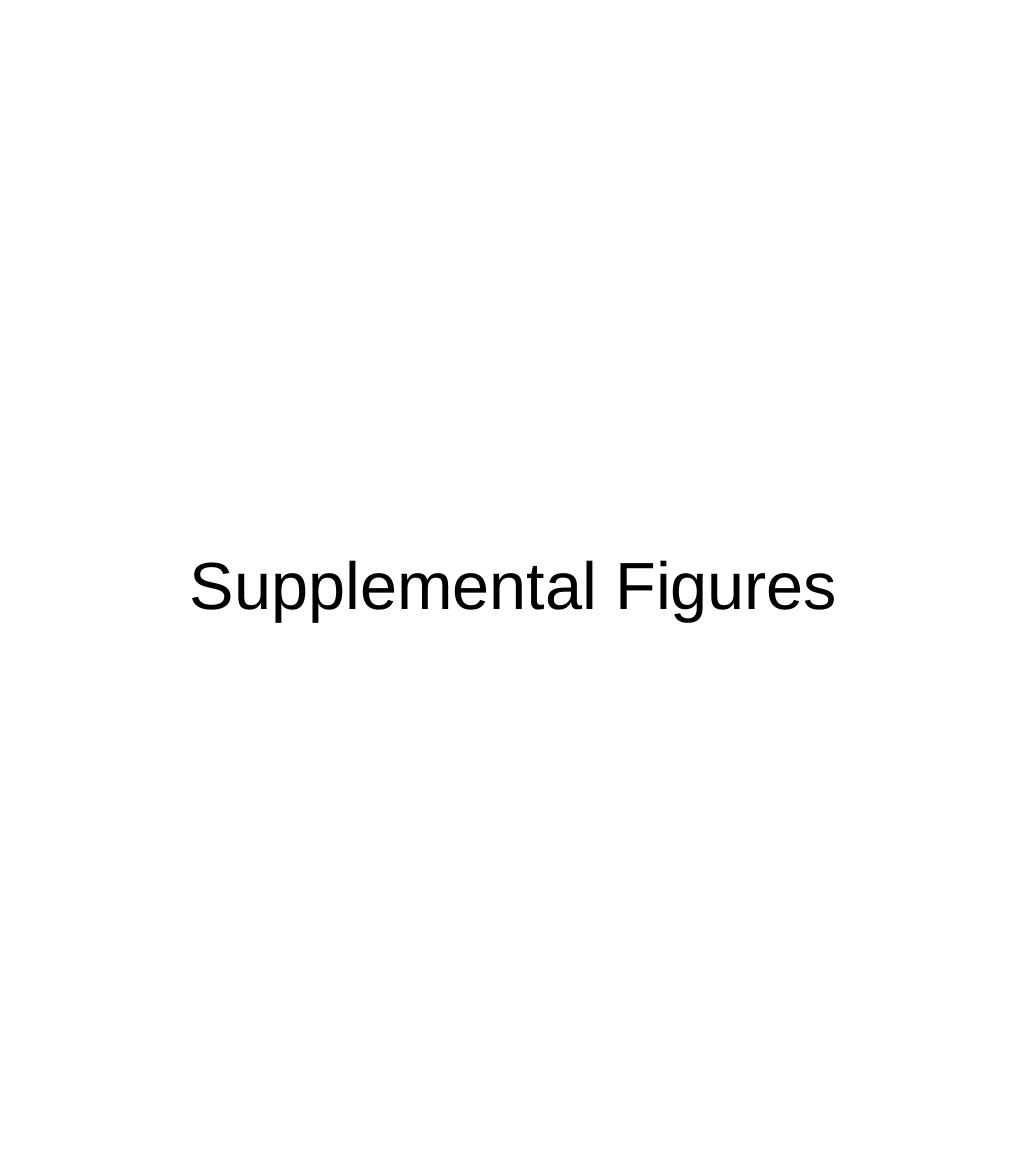

Supplemental Figures

## Slide 3
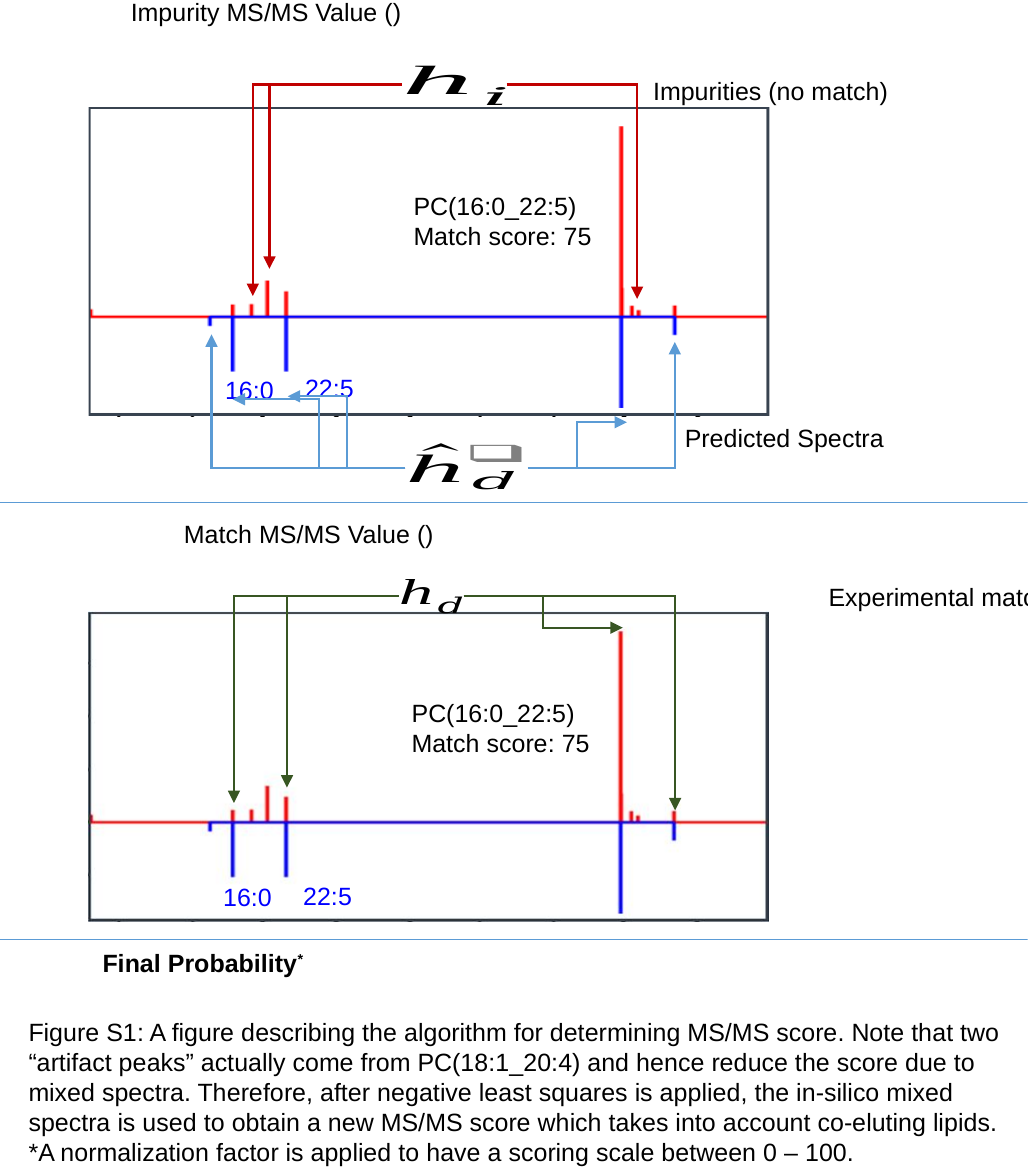

Impurities (no match)
PC(16:0_22:5)
Match score: 75
22:5
16:0
Predicted Spectra
Experimental matched spectra
PC(16:0_22:5)
Match score: 75
22:5
16:0
Figure S1: A figure describing the algorithm for determining MS/MS score. Note that two “artifact peaks” actually come from PC(18:1_20:4) and hence reduce the score due to mixed spectra. Therefore, after negative least squares is applied, the in-silico mixed spectra is used to obtain a new MS/MS score which takes into account co-eluting lipids. *A normalization factor is applied to have a scoring scale between 0 – 100.

## Slide 4
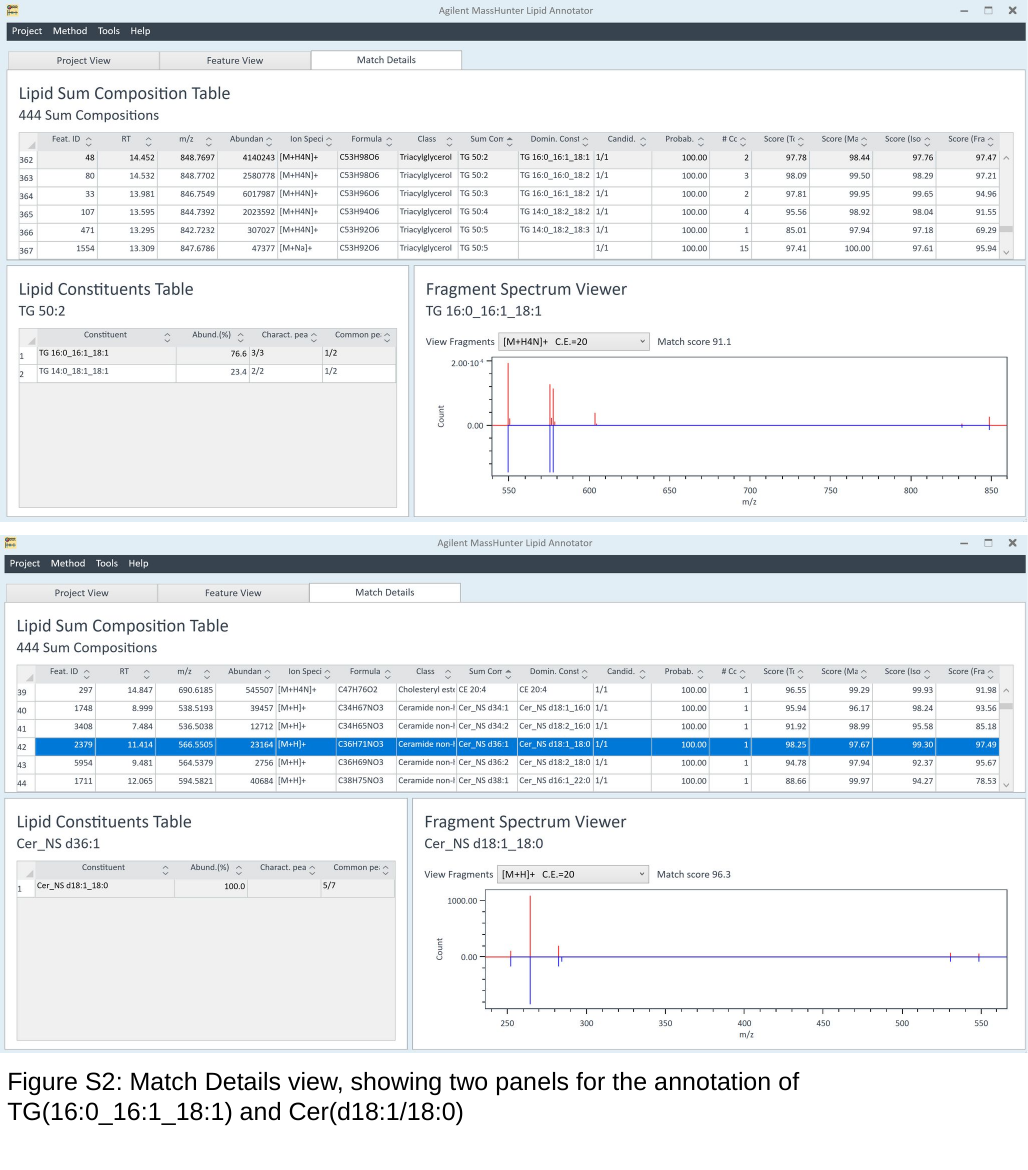

Figure S2: Match Details view, showing two panels for the annotation of TG(16:0_16:1_18:1) and Cer(d18:1/18:0)

## Slide 5
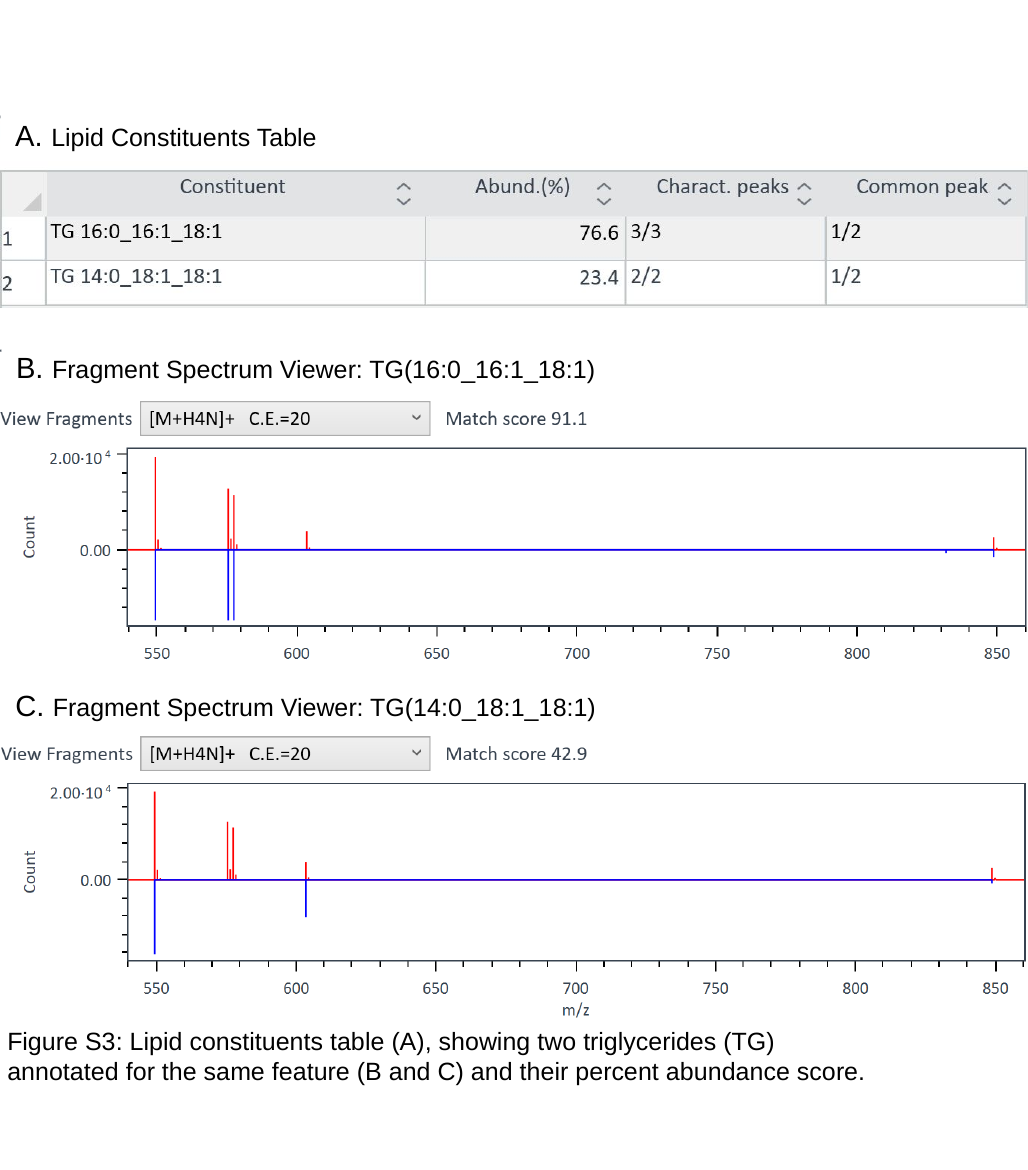

A. Lipid Constituents Table
B. Fragment Spectrum Viewer: TG(16:0_16:1_18:1)
C. Fragment Spectrum Viewer: TG(14:0_18:1_18:1)
Figure S3: Lipid constituents table (A), showing two triglycerides (TG) annotated for the same feature (B and C) and their percent abundance score.

## Slide 6
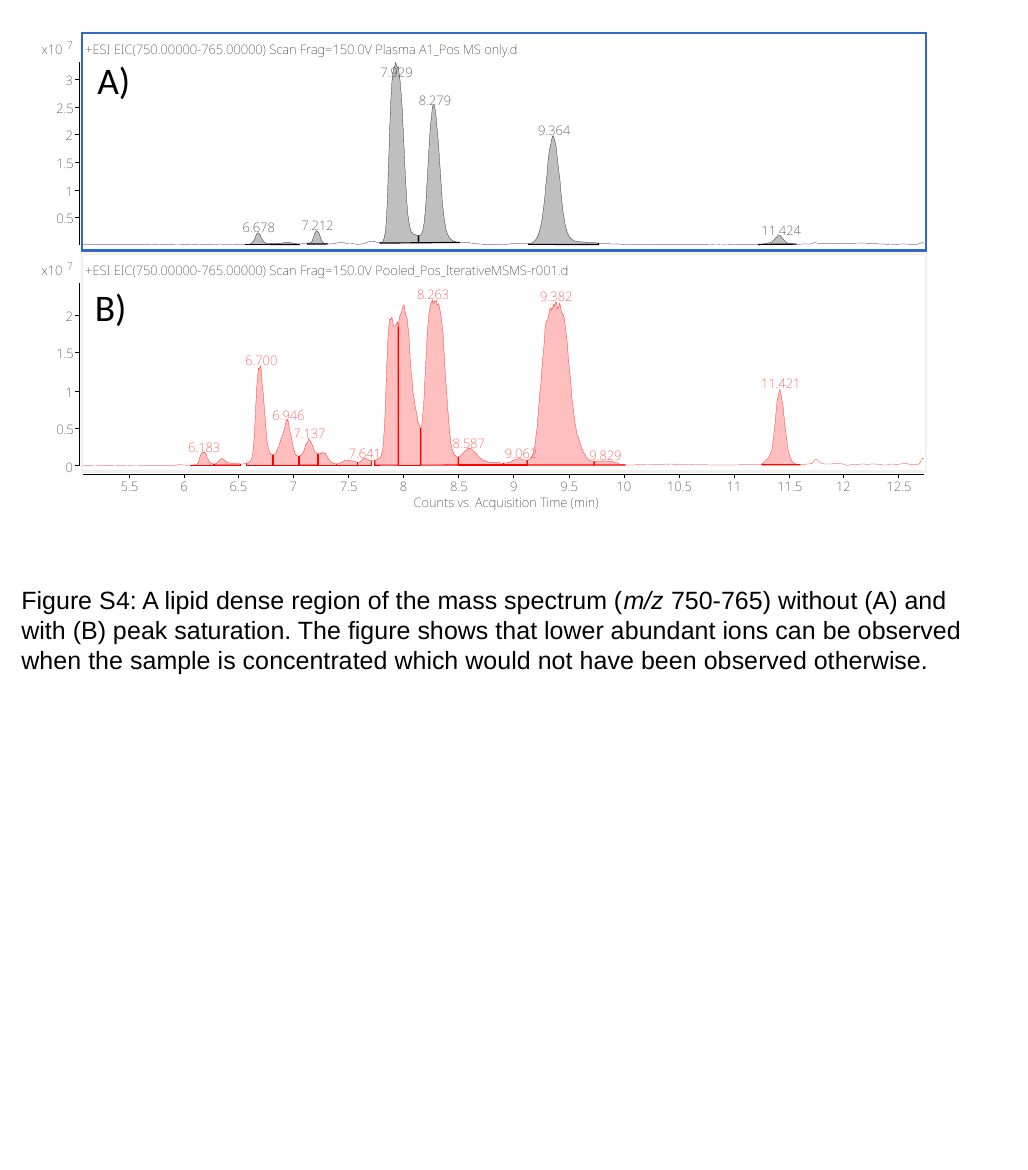

A)
B)
Figure S4: A lipid dense region of the mass spectrum (m/z 750-765) without (A) and with (B) peak saturation. The figure shows that lower abundant ions can be observed when the sample is concentrated which would not have been observed otherwise.

## Slide 7
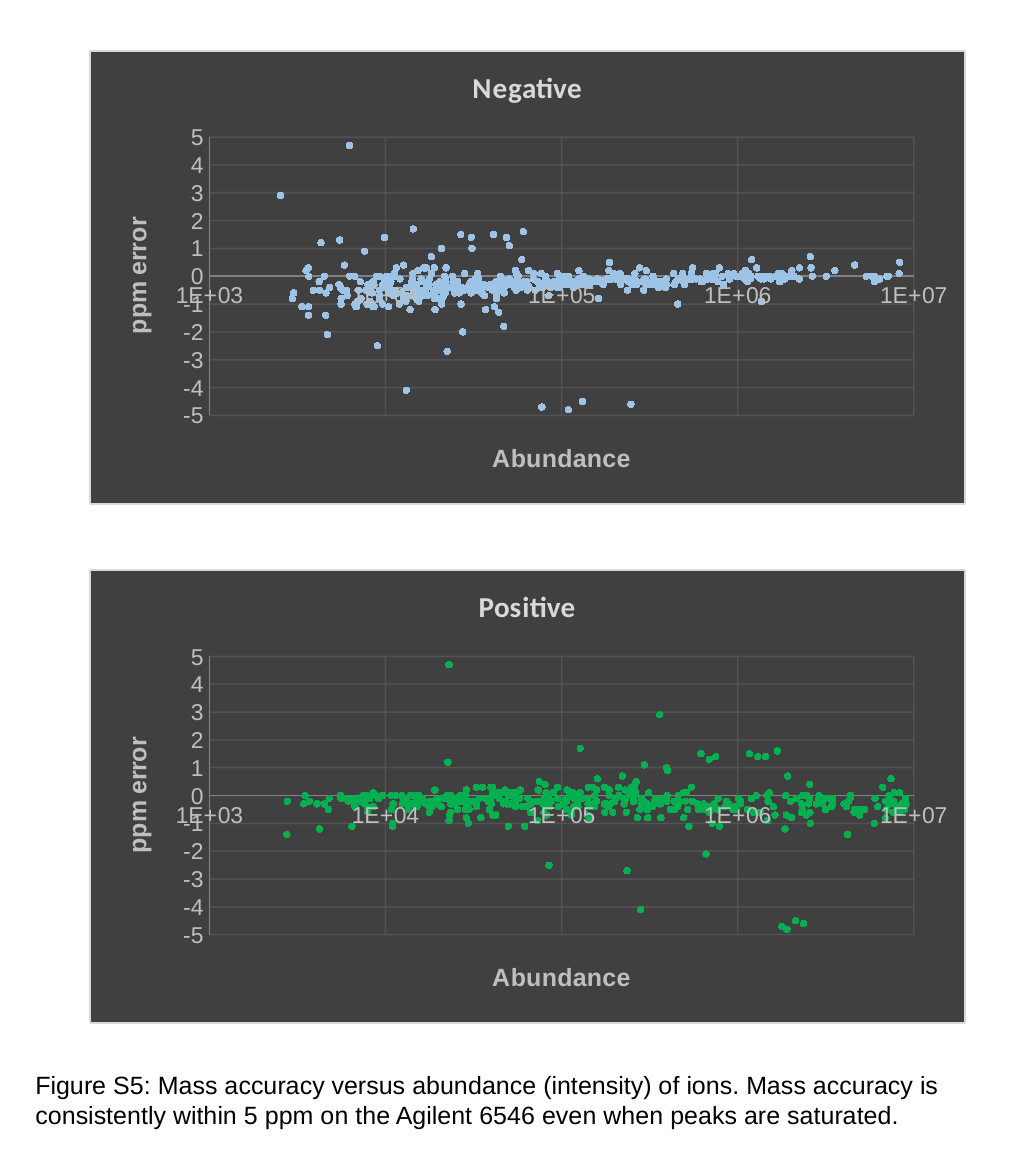

### Chart:
| Category | |
|---|---|
### Chart:
| Category | |
|---|---|Figure S5: Mass accuracy versus abundance (intensity) of ions. Mass accuracy is consistently within 5 ppm on the Agilent 6546 even when peaks are saturated.

## Slide 8
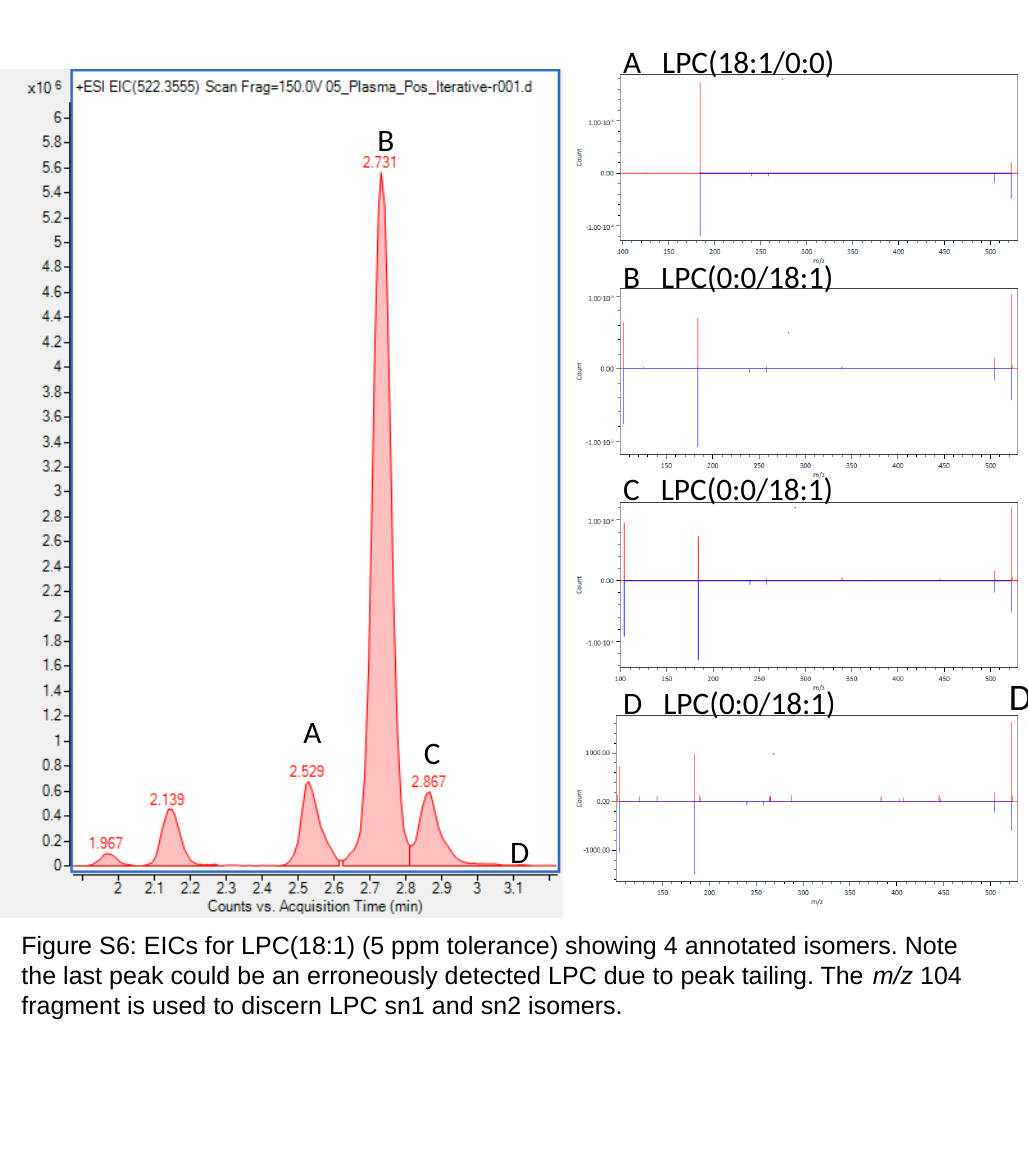

A LPC(18:1/0:0)
B
B LPC(0:0/18:1)
C LPC(0:0/18:1)
D: may be tail of previous peak
D LPC(0:0/18:1)
A
4
+ESI EIC(522.3555) Scan Frag=150.0V 05_Plasma_Pos_Iterative-r001.d
x10
2.6
2.4
2.2
2
1.8
1.6
1.4
1.2
1
0.8
0.6
0.4
0.2
2.97
2.98
2.99
3
3.01
3.02
3.03
3.04
3.05
3.06
3.07
3.08
3.09
3.1
3.11
3.12
3.13
3.14
3.15
3.16
3.17
3.18
3.19
Counts vs. Acquisition Time (min)
C
D
Figure S6: EICs for LPC(18:1) (5 ppm tolerance) showing 4 annotated isomers. Note the last peak could be an erroneously detected LPC due to peak tailing. The m/z 104 fragment is used to discern LPC sn1 and sn2 isomers.

## Slide 9
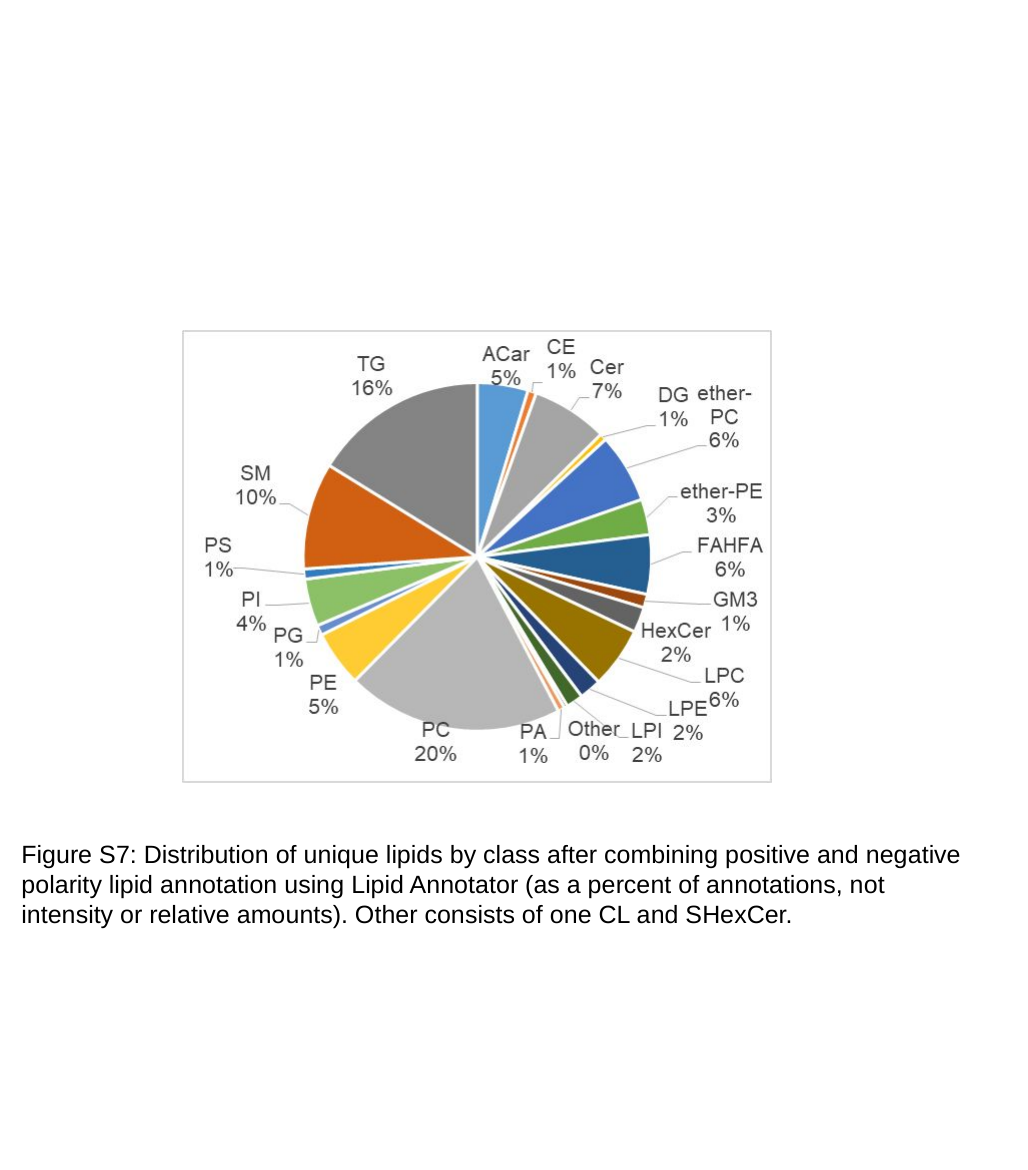

Figure S7: Distribution of unique lipids by class after combining positive and negative polarity lipid annotation using Lipid Annotator (as a percent of annotations, not intensity or relative amounts). Other consists of one CL and SHexCer.

## Slide 10
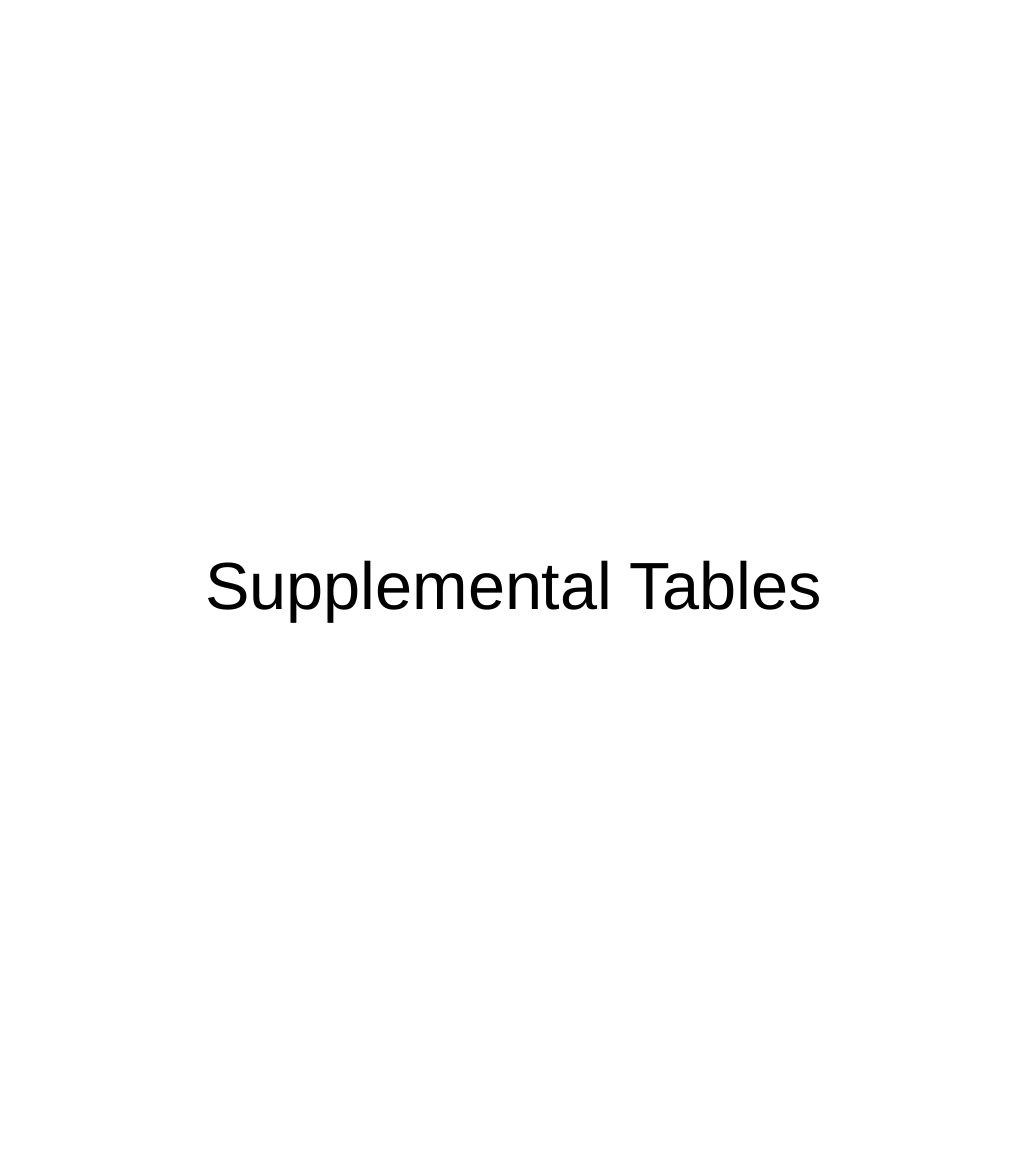

Supplemental Tables

## Slide 11
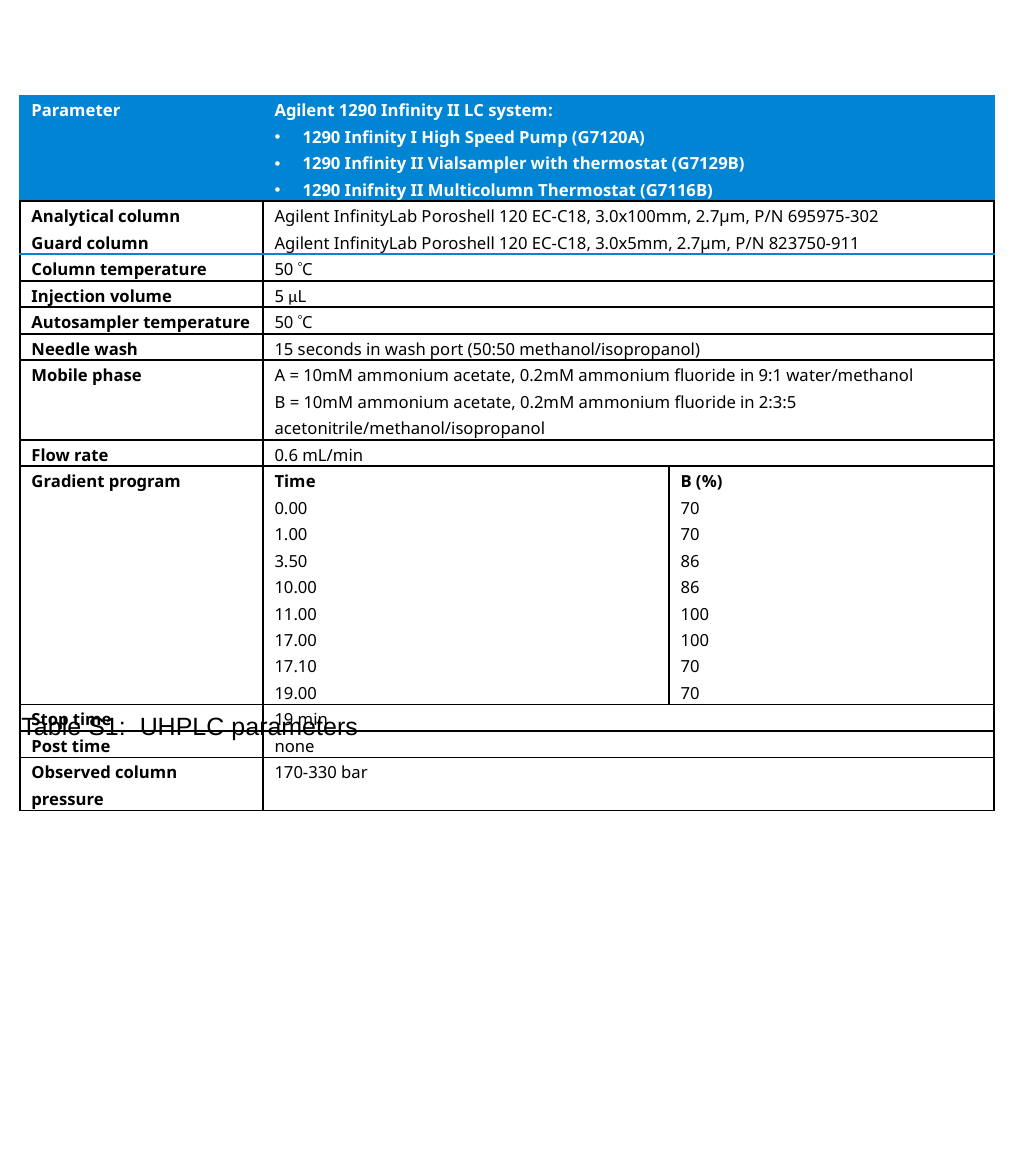

| Parameter | Agilent 1290 Infinity II LC system: 1290 Infinity I High Speed Pump (G7120A) 1290 Infinity II Vialsampler with thermostat (G7129B) 1290 Inifnity II Multicolumn Thermostat (G7116B) | |
| --- | --- | --- |
| Analytical column Guard column | Agilent InfinityLab Poroshell 120 EC-C18, 3.0x100mm, 2.7µm, P/N 695975-302 Agilent InfinityLab Poroshell 120 EC-C18, 3.0x5mm, 2.7µm, P/N 823750-911 | |
| Column temperature | 50 °C | |
| Injection volume | 5 µL | |
| Autosampler temperature | 50 °C | |
| Needle wash | 15 seconds in wash port (50:50 methanol/isopropanol) | |
| Mobile phase | A = 10mM ammonium acetate, 0.2mM ammonium fluoride in 9:1 water/methanol B = 10mM ammonium acetate, 0.2mM ammonium fluoride in 2:3:5 acetonitrile/methanol/isopropanol | |
| Flow rate | 0.6 mL/min | |
| Gradient program | Time 0.00 1.00 3.50 10.00 11.00 17.00 17.10 19.00 | B (%) 70 70 86 86 100 100 70 70 |
| Stop time | 19 min | |
| Post time | none | |
| Observed column pressure | 170-330 bar | |
Table S1: UHPLC parameters

## Slide 12
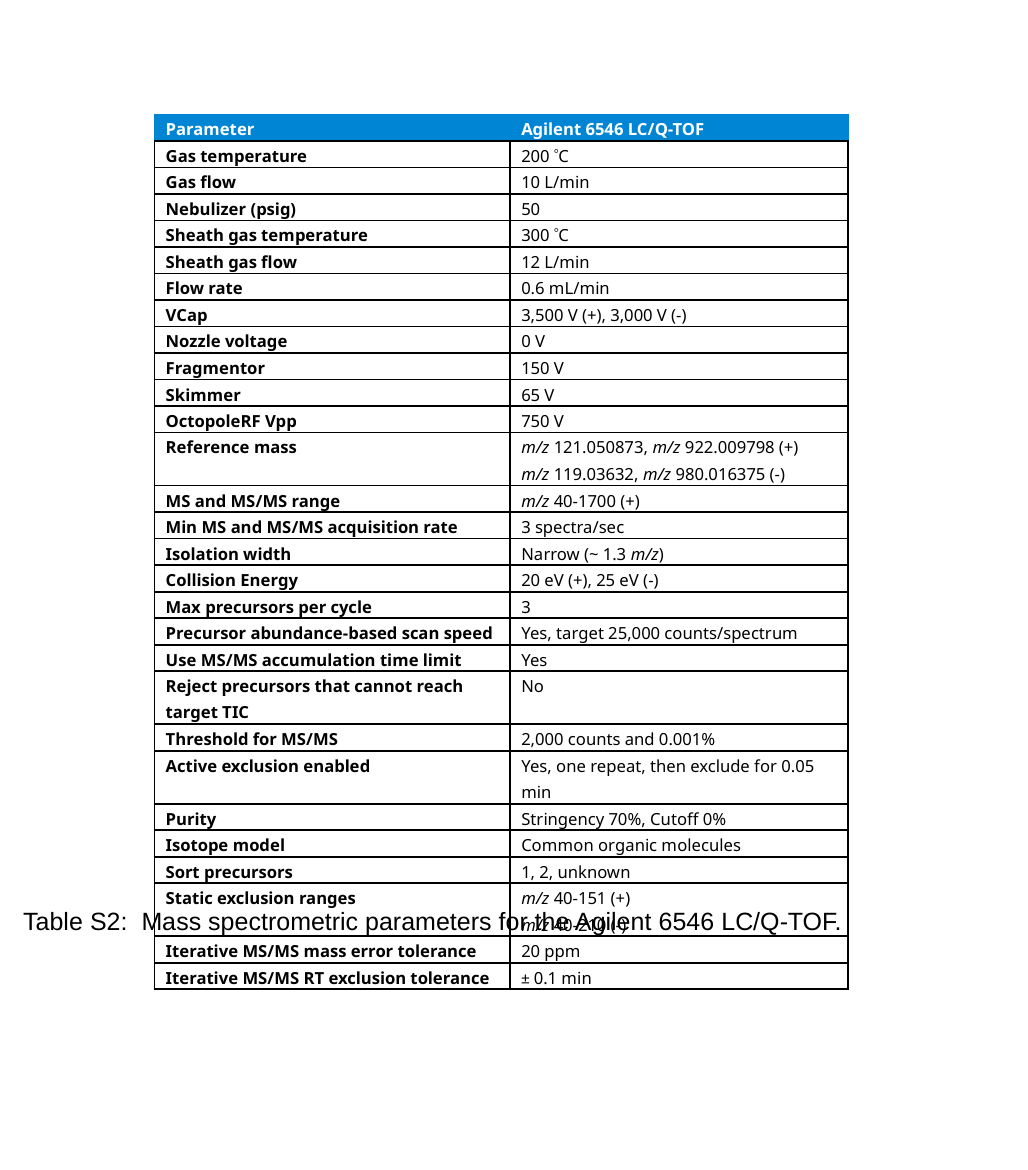

| Parameter | Agilent 6546 LC/Q-TOF |
| --- | --- |
| Gas temperature | 200 °C |
| Gas flow | 10 L/min |
| Nebulizer (psig) | 50 |
| Sheath gas temperature | 300 °C |
| Sheath gas flow | 12 L/min |
| Flow rate | 0.6 mL/min |
| VCap | 3,500 V (+), 3,000 V (-) |
| Nozzle voltage | 0 V |
| Fragmentor | 150 V |
| Skimmer | 65 V |
| OctopoleRF Vpp | 750 V |
| Reference mass | m/z 121.050873, m/z 922.009798 (+) m/z 119.03632, m/z 980.016375 (-) |
| MS and MS/MS range | m/z 40-1700 (+) |
| Min MS and MS/MS acquisition rate | 3 spectra/sec |
| Isolation width | Narrow (~ 1.3 m/z) |
| Collision Energy | 20 eV (+), 25 eV (-) |
| Max precursors per cycle | 3 |
| Precursor abundance-based scan speed | Yes, target 25,000 counts/spectrum |
| Use MS/MS accumulation time limit | Yes |
| Reject precursors that cannot reach target TIC | No |
| Threshold for MS/MS | 2,000 counts and 0.001% |
| Active exclusion enabled | Yes, one repeat, then exclude for 0.05 min |
| Purity | Stringency 70%, Cutoff 0% |
| Isotope model | Common organic molecules |
| Sort precursors | 1, 2, unknown |
| Static exclusion ranges | m/z 40-151 (+) m/z 40-210 (-) |
| Iterative MS/MS mass error tolerance | 20 ppm |
| Iterative MS/MS RT exclusion tolerance | ± 0.1 min |
Table S2: Mass spectrometric parameters for the Agilent 6546 LC/Q-TOF.

## Slide 13
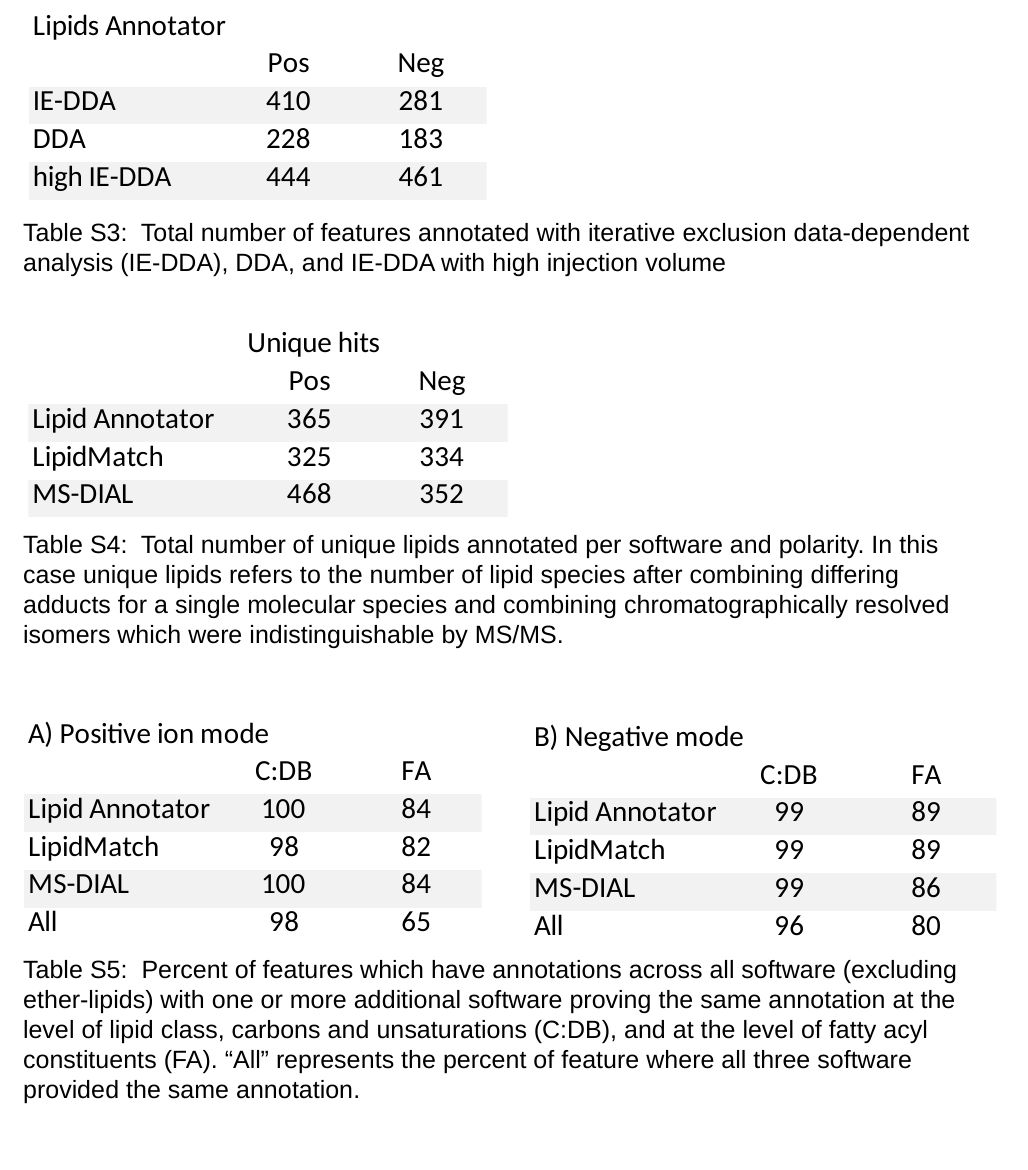

Table S3: Total number of features annotated with iterative exclusion data-dependent analysis (IE-DDA), DDA, and IE-DDA with high injection volume
Table S4: Total number of unique lipids annotated per software and polarity. In this case unique lipids refers to the number of lipid species after combining differing adducts for a single molecular species and combining chromatographically resolved isomers which were indistinguishable by MS/MS.
Table S5: Percent of features which have annotations across all software (excluding ether-lipids) with one or more additional software proving the same annotation at the level of lipid class, carbons and unsaturations (C:DB), and at the level of fatty acyl constituents (FA). “All” represents the percent of feature where all three software provided the same annotation.
